# Dental practitioner recruitment for a randomized clinical trial in the field to evaluate the performance of a new glass ionomer restoration material

**DOI:** 10.1186/s13063-016-1198-3

**Published:** 2016-02-10

**Authors:** Thomas Klinke, Amro Daboul, Christian Schwahn, Roland Frankenberger, Reinhard Hickel, Reiner Biffar

**Affiliations:** Policlinic of Prosthodontics and Biomaterials, Greifswald University, Greifswald, Germany; Conservative Dentistry Department, Philipps University of Marburg, Marburg, Germany; Policlinic for Restorative Dentistry and Periodontology, University of Munich, Munich, Germany

**Keywords:** Dental restoration, glass ionomer, multicenter study, personnel recruitment

## Abstract

**Background:**

In 2009, we began recruiting dental practitioners across Germany to participate in a clinical trial to evaluate the clinical performance of EQUIA, a new glass ionomer restoration material. The aim of this paper is to discuss the outcomes of the dental practitioner recruitment and outline the process of establishing a practice-based research network.

**Methods:**

Study proposals were sent to randomly selected dental offices in 29 cities in Germany. The proposals were sent until a minimum of 10 clinics in each city declared participation. Later on, briefing lectures informed the participating practitioners about the design, methods, and material application procedure. Participants were familiarized with the guidelines of Good Manufacturing Practice (GMP) and Good Epidemiological Practice (GEP). A questionnaire describing the characteristics of each dental office was filled out by the participating practitioner. Additionally, participation levels were characterized according to the socioeconomic status and geographic districts of residence in Germany (Regions 0 to 9). The associations between the characteristics were tested by the Kruskal-Wallis Test and Chi-squared test (*P* < 0.05).

**Results:**

A total of 3194 private dental clinics were invited, 1712 clinics refused to participate, 1195 did not respond to the invitation, and 323 agreed to participate. Only 144 clinics participated in the lectures held in their cities and signed the participation agreement. Based on their geographic location, the highest participation was in Region 2 with a participation rate of 14.3 %, and the lowest participation was in Region 6 with a participation rate of 1.7 %. Regions with the lowest rate of unemployment and relatively higher rates of income (Regions 7 and 8) had the highest rate of refusals (86 %).

**Conclusion:**

The initial results of the dental practitioner recruitment in this study suggest that the recruitment and pre-randomization design were successful, and by reaching out to a considerable number of private dental clinics to participate, we were able to recruit a smaller number of highly motivated dentists in this clinical study. Regional differences in socioeconomic status, practitioner specialization, and differences in patient health care insurance have to be considered when recruiting dental practitioners for clinical trials.

**Trial registration:**

The trial has been registered at Deutsches Register Klinischer Studien (German register of clinical trials) on 6 September 2012 under DRKS-ID: DRKS00004220.

**Electronic supplementary material:**

The online version of this article (doi:10.1186/s13063-016-1198-3) contains supplementary material, which is available to authorized users.

## Background

Randomized clinical trials (RCTs) are widely recognized for providing important scientific evidence on the efficacy and safety of new therapies, materials, and techniques in the dental field. Likewise, researchers need to design clinical trials to demonstrate differences between newer therapies or materials and existing ones. The choice of which kind of study design will be used is influenced by scientific findings, patient considerations, and the outcome application [1–4]. A clinical trial might have either a prospective or a retrospective design. Whereas the data or the outcome of interest has already been collected at the start of a retrospective study, and important data often may not be available, a prospective study design would allow collection of data at regular time intervals and follows the outcome of interest in a forward direction. Although the strength of the evidence obtained from prospective studies, is higher than from retrospective studies, data collection in prospective studies is more complex, time consuming, and expensive. Furthermore, the required large sample sizes and potential losses to follow-up can present further problems in prospective studies and create substantial difficulties compared to other types of analytical studies.

Because of the complexity of prospective RCTs in each project phase (organization, initiation, planning, implementation, control, and finalizing), RCTs are mostly carried out as single-center studies (SCS) in hospitals or other academic institutions, which have access to specific patient cohorts, special trained clinicians, and can control time and other confounding factors [5]. To increase the reliability of data extracted from clinical trials, researchers have to extend their clinical trials to include general dental practice rather than restrict their trials to dental schools or centers. Such practice-based dental research networks have existed in the United Kingdom and United States for a long time [6–9] and have become important for the dental research community, particularly in clinical trials on restorative dentistry [10–13]. The incorporation of dental practitioners into research on new materials or techniques would not only benefit the research by reflecting the real-world situation on treatment outcomes, it would also reduce the bias and influence experienced in single-center studies [14].

Since its development in the 1970s, glass ionomer cements (GIC) have been widely used as a restorative material for anterior teeth and no-load bearing surfaces, mainly in Class III and V cavities. Nonetheless, GIC is considered a semipermanent restoration material for Class I and Class II cavities in permanent teeth, and the use of GIC as a permanent restoration material is often questioned because of its poor wear resistance and tensile and flexural strengths, which would result in a higher rate of early fractures compared to amalgam and composite materials. In recent years, two encapsulated glass ionomers, for which the manufacturer claims high mechanical properties, have been marketed. These include a fast-setting conventional GIC coated with an unfilled resin coating (LC coated Fuji IX, GC, Tokyo, Japan) and a fast- setting GIC with a nano-filled resin coat (Equia system, GC, Tokyo, Japan), which is supposed to have an increased wear resistance and can be used as a replacement for amalgam and composite fillings in class I and II cavities.

Until now, clinical studies on the performance of GIC fillings have been limited to 1) retrospective studies performed on conventional or enforced GIC, 2) studies evaluating GIC fillings placed with an atraumatic restorative treatment (ART) technique, or 3) short-term prospective clinical trials on GIC under ideal conditions in university environments.

In 2009, we began recruiting dental practitioners in their private dental clinics across Germany to participate in a clinical trial intended to evaluate the clinical performance of EQUIA, the new GIC with a nano-filled coating, as a permanent restoration in class I and II cavities. Therefore, the purpose of this paper is to discuss the outcomes of dental practitioner recruitment and outline the process of establishing a practice-based network to evaluate the clinical performance of the Equia system and LC-coated GIC.

## Methods

A double-blinded, randomized, prospective clinical trial in the field was primarily designed to assess the clinical performance of two variants of a commonly used dental material promoted as an alternative filling material to amalgam and composite in posterior teeth. The recruitment process of dentists and patients from private dental clinics was evaluated and characterized according to the socioeconomic status and geographic districts of residence in Germany.

To insure statistical power, the minimal representative sample size was based on the required number of fillings to evaluate both materials (n = 440 for each group). The homogeneity of the participating clinics was guaranteed by the recruitment and the criterion that only one or two fillings will be placed for each patient. Therefore, an exponential maximum likelihood test of equality with a *P* = 0.05 two-sided significance level will have 90 % power to detect a difference between the Group 1 (Equia fillings) exponential parameter, γ1 of 20 % 0.021 (corresponding to a proportion of 30 % after 60 months), and the Group 2 (LC-coated Fuji IX) exponential parameter, γ2 of 0.026 (corresponding to a proportion of 20 % after 60 months). This test will involve a constant hazard ratio 0.021/0.026 = 0.75 and assume an accrual period of 12 months, a maximum follow-up time of 60 months (5 years), and a common exponential dropout rate of 1 %.

### Dental practitioner recruitment

**Fig. 1:**
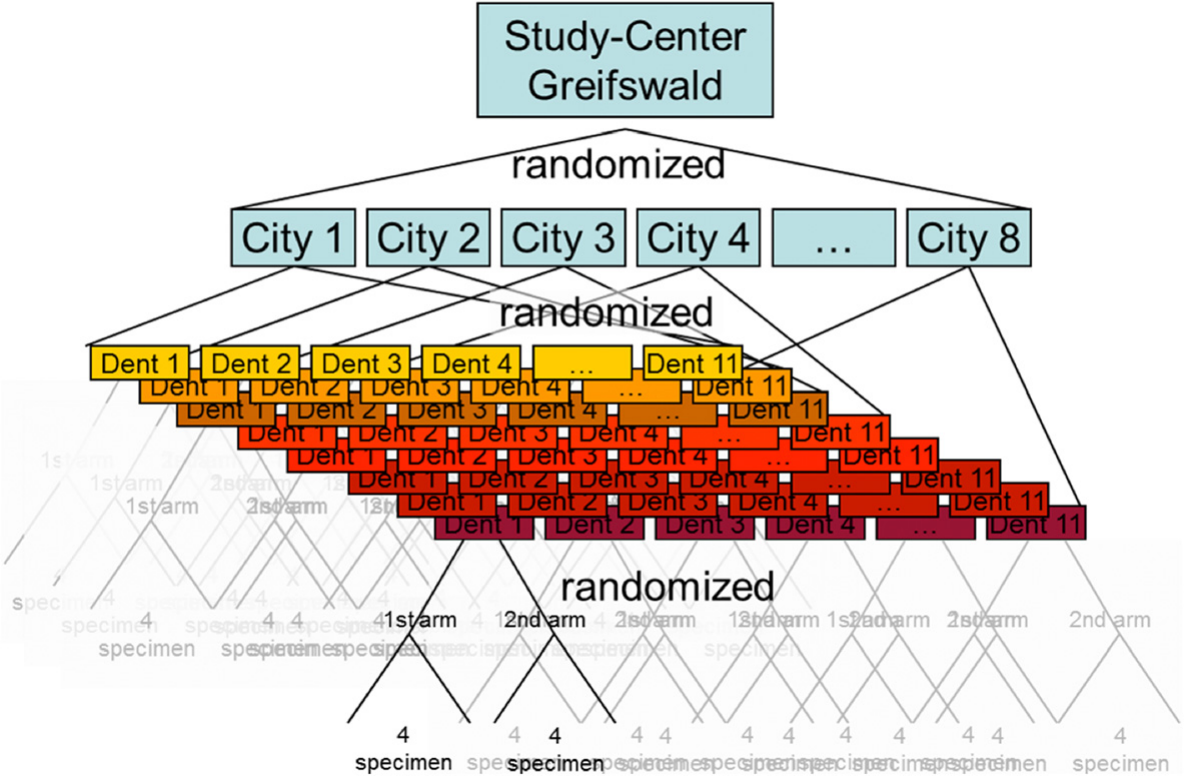
Levels of randomization of the cities, dental clinics, and distributed glass ionomer cement (GIC) filling material

**Fig. 2:**
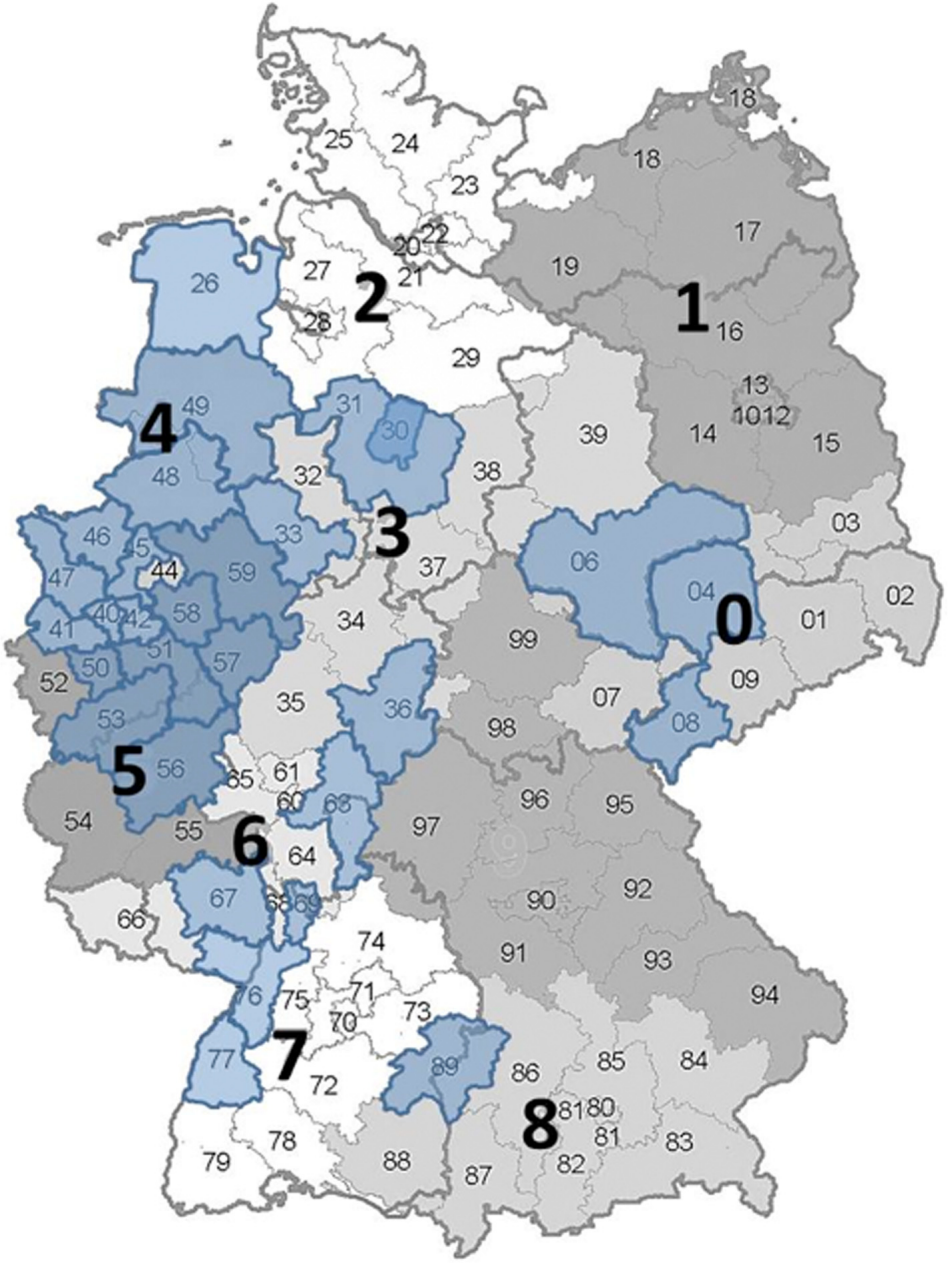
Location of the invited dental practices according to the working zip code areas in Germany

The dental practitioner recruitment process began in September 2009 and was completed in July 2011. The recruitment was carried out in a two-stage design (Fig. 1). First, German cities with 50 to 250 thousand inhabitants were identified and then randomized (n = 164). After randomization, one city at a time was selected, and private dental clinics in the city were identified and then randomized. The recruitment began when individually addressed cover letters with a short study proposal, the aim and hypothesis of the study, and details on what participation would involve were mailed. The proposals were sent to 30 randomly selected dental offices at a time, and in cases where the advised number of participating offices was not reached, other clinics in the city were invited until a minimum of 10 clinics declared their intention to participate. When no decision (agreement or refusal) was received from an invited clinic, a telephone call was placed asking for confirmation; in the case of refusal, the reason for refusal was asked, and the dental clinic was deleted from the mailing list. At the end of the recruitment process, 3194 invitations had been issued, covering 29 randomly selected cities in Germany. The locations of the invited offices (ZIP-Code region) are shown in Fig. 2.

Later, the data on the socioeconomic status (SES) of each region were used to describe the participating randomly selected local communities [15], and the following surrogate variables were considered: a) the inhabitants per square kilometers; b) household status (one-person households or multi-persons households); c) monthly income per household (low, above 1000€ and less than 4000€, or high, above 4000€); and d) the rate of unemployment. The associations between the variables were tested by the Kruskal-Wallis (U-) Test and Chi-squared test (*P* < 0.05).

### Patients’ recruitment

In the dental clinics, the patients were asked to participate in the study only when a dental filling was indicated on posterior teeth. Patients who agreed to participate had to sign an informed consent of agreement according to Good Clinical Practice (GCP) and the Declaration of Helsinki. Patients who were not interested in participation were asked for the reason and filled in a short questionnaire (three items: education, dental attendance, and last visit to a dentist).

Each participant got a unique pseudonym in the dental practice, and the unique pseudonym key remained in the patient’s list at the dental practice.

### Calibration and education

A lecture held in each participating city educated and familiarized the participating dentists with the internationally approved directives for clinical trials according to Good Clinical Practice (GCP). Participating dentists were also briefly informed on the specific processing methodology of Good Manufacturing Practice (GMP) and Good Epidemiological Practice (GEP). The study operating procedure was then discussed, and the precise clinical trial conditions were given in printed form to each participant. At the end of the lecture, a certificate of participation was issued to each participating dentist, along with a questionnaire describing the characteristics of the participating dental office (office location, number of dentists, age of dental practice, dentist speciality, number of patients with statutory health insurance, and the number of privately insured patients). The associations between the characteristics were tested by the Kruskal-Wallis Test and Chi-squared test (*P* < 0.05).

Later, each participating dental practice received a package by mail with 10 de-identified and re-labelled filling capsules with their coatings (five re-labelled boxes of the Equia system as label A and five re-labelled boxes of LC-coated Fuji IX as label B), so neither the dental practitioner nor the patient knew which material was used in this blinded design.

Each finished filling was given a pseudonym consisting of four digital fields: 1) practitioner ID, 2) patient pseudonym, 3) cavity class, and 4) material label.

Finally, all pseudonymized information that was collected from the participating dentists and patients was stored and monitored by a special committee. The Data Safety and Monitoring Committee (DSMC) based in Munich University will guarantee the abidance of randomization and the quality of the data acquisition and database.

This clinical trial was approved by the ethical commission at Greifswald University (No.: BB 33/09).

## Results

Table 1 presents the results of the recruitment that began in September 2009 of the private dental clinics. A total of 3194 private dental clinics were invited, of which 53.6 % (n = 1712) refused to participate, 36.3 % (n = 1159) did not respond to the invitation, and 10.1 % (n = 323) agreed to participate.

**Table 1:** Results of the recruitment of the private dental clinics

		Trial participation					Location of the GDP		
ZIP code region	Study proposal (N = 3194)	Refusal	Open decision	Promise	Lecture participation (N = 144)		Urban	Outskirts of town	Rural
		[%]	[%]	[%]	N	[%]	[%]	[%]	[%]
0	396	14.6	77.5	7.8	15	7,8	53.3	40.0	6.7
2	70	62.9	12.9	25.7	10	14.3	60.0	30.0	10.0
3	294	56.5	32.0	11.9	27	9.2	77.8	14.8	3.7
4	471	80.9	11.9	7.6	24	5.5	58.3	33.3	8.3
5	738	42.8	56.2	7.0	42	5.8	54.8	28.6	11.9
6	588	31.3	71.3	3.6	8	1.7	87.5	12.5	0
7/8^a^	637	86.0	0.3	6.9	18	5.2	61.1	22.2	16.7
	MEAN [%]	53.6	37.4	10.1		7.1	64.7	25.9	8.2

^a^Because of the close location, Regions 7 and 8 were calculated together

Of the 323 clinics that agreed to participate, only 144 clinics (44.6 %) (4.5 % of the invited clinics, mean 7.1 % of invited clinics in all regions) participated in the lectures held in their cities and signed the participation agreement.

The majority of the participating dental practitioners were specialized. That is, 31 % were specialized in periodontology, 20 % in pediatric dentistry, 9 % in oral surgery, and 9 % in orthodontics. In total, 60 % of the dental practices were operated by one practitioner, 30 % had two dentists, and 10 % had three or more practitioners.

Most of the participating dental clinics were located in the urban parts of towns (64.7 % n = 94), 25.9 % (n = 37) were located in the outskirts, and 8.2 % (n = 13) were located in rural areas (Table 1).

Based on their geographic location, the highest participation was in Region 2, with a participation rate of 14.3 %, and the lowest participation was in Region 6, with a participation rate of 1.7 %. Regions with the lowest rate of unemployment and relatively higher rates of income (Regions 7 and 8) had the highest rate of refusals (86 %) in trial participation (Table 2).

**Table 2:** Socioeconomic status in the recruitment regions

Zip code		Inhabitants per km^2^	One-person household with no children [%]	Multi-person household with children [%]	Household income (< €1 T) [%]	Household income (< €4 T) [%]	Rate of unemployment [%]
0	Mean (SD)	1129.80 (810.62)	33.52 (12.01)	23.34 (1,21)	18.90 (0.5)	9.18 (0.91)	15.82 (1.81)
	SE	209.30	3.10	0.31	0.13	0.24	0.47
	Minimum	171.0	19.3	21.9	18.5	8.0	13.7
	Maximum	1788.0	43.0	24.3	19.5	10.0	17.5
*P* value (¥/†)		…/n.s.	…/n.s.	…/n.s.	…/n.s.	…/n.s.	…/n.s.
2	Mean (SD)	211.60 (130.42)	36.74 (2.11)	32.27 (1.54)	13.33 (1.81)	16.46 (2.37)	9.82 (3.37)
	SE	41.24	0.67	0.49	0.57	0.75	1.06
	Minimum	146.0	33.3	30.8	9.9	15.0	6.8
	Maximum	459.0	38.5	33.8	14.3	21.0	14.7
	*P* value (¥/†)	<0.0001/n.s.	n.s./n.s.	<0.0001/n.s.	<0.0001/n.s.	<0.0001/n.s.	<0.0001/n.s.
3	Mean (SD)	475.80 (377.99)	35.60 (4.84)	34.71 (4.93)	11.64 (1.76)	17.85 (1.82)	8.39 (2.18)
	SE	69.01	0.88	0.89	0.32	0.33	0.39
	Minimum	109.0	32.1	26.0	8.8	16.0	6.4
	Maximum	1104.0	44.8	39.3	14.4	21.0	12.3
	*P* value (¥/†)	<0.01/n.s.	n.s./n.s.	<0.0001/n.s.	<0.0001/n.s.	<0.0001/n.s.	<0.0001/n.s.
4	Mean (SD)	636.50 (327.17)	34.26 (1.90)	33.09 (0.43)	9.41 (1.55)	21.36 (2.25)	9.63 (1.00)
	SE	71.39	0.41	0.43	0.33	0.49	0.21
	Minimum	210.0	29.5	30.1	7.8	17.0	8.5
	Maximum	1469.0	39.6	37.6	12.9	24.0	11.6
	*P* value (¥/†)	n.s./n.s.	n.s./n.s.	<0.0001/n.s.	<0.0001/n.s.	<0.0001/n.s.	<0.0001/n.s.
5	Mean (SD)	418.49 (204.16)	33.88 (1.98)	33.30 (1.41)	10.35 (1.13)	19.85 (2.12)	9.58 (1.13)
	SE	31.13	0.30	0.21	0.17	0.32	0.17
	Minimum	195.0	30.8	27.9	8.1	15.0	7.4
	Maximum	1175.	43.9	38.1	13.8	24.0	11.3
	*P* value (¥/†)	n.s./n.s.	n.s./<0.01	<0.0001/<0.0001	<0.0001/<0.01	<0.0001/<0.0001	<0.0001/<0.001
6	Mean (SD)	810.20 (323.78)	36.22 (0.62)	31.28 (1.52)	9.20 (1.56)	23.14 (2.81)	8.60 (0)
	SE	144.80	0.28	0.68	0.70	1.26	0.10
	Minimum	231.0	35.1	30.6	8.5	18.0	8.6
	Maximum	955.0	36.5	34.0	12.0	24.0	8.7
	*P* value (¥/†)	n.s./n.s.	n.s./n.s.	<0.0001/n.s.	<0.0001/n.s.	<0.0001/n.s.	<0.01/n.s.
7/8^a^	Mean (SD)	560.30 (433.38)	37.56 (4.22)	31.44 (5.27)	11.10 (2.91)	18.31 (3.61)	6.75 (2.91)
	SE	96.90	0.94	1.18	0.65	0.80	0.65
	Minimum	134.0	32.4	21.9	7.6	8.0	2.8
	Maximum	1375.0	43.9	38.7	19.5	23.0	11.0
	*P* value (¥/†)	<0.01/n.s.	n.s./n.s.	<0.0001/n.s.	<0.0001/n.s.	<0.0001/n.s.	<0.0001/n.s.

^a^Because of the close location, these regions were combined for the calculations

¥Wilcoxon-Mann-Whitney test, U-Test, *P* < 0.05

†Chi-squared test, *P* < 0.05

## Discussion

Most of the research performed on dental materials, including on glass ionomer cements, is carried out in dental schools or other academic institutions, rather than in general dental practice where the majority of dental treatment is performed. The majority of trials on new dental materials and methods are usually conducted in a parallel randomized controlled design as single-center studies; therefore, they are more prone to bias and may have lower methodological quality than multicenter studies [14, 16]. To obtain results that reflect the real world or the outside dental treatment routine, it is necessary to implement private dental clinics in clinical trials [6]. Moreover, previous literature has indicated that including private practitioners and their daily routine environments in dental studies would increase the practitioners’ acceptance for equivalence studies [16–18]. Another advantage of multicenter practice-based studies is the inclusion of larger cohorts with demographic characteristics that might influence the dental treatment. Nevertheless, such expansions are accompanied by higher expenses and require more time for the recruitment of dental practitioners and any planned follow-ups.

### Dental practitioner recruitment

Our initial experience was that recruitment was proceeding slowly and that a combination of different recruitment strategies was necessary [19]. We were able to increase the recruitment rate by following a combination of approaches in our recruitment process that included written letters, follow-up telephone calls, and visits to the dental clinics. We also followed the recommendations set by a recent systematic review [20] on enhancing the response rate by personalizing the letters of invitation and using user-friendly questionnaires with stamped return envelops. Other recommendations to increase the participation rate were offering continuous medical education points and avoiding the change of the research personnel in contact with the dental practitioners [21].

**Fig. 3:**
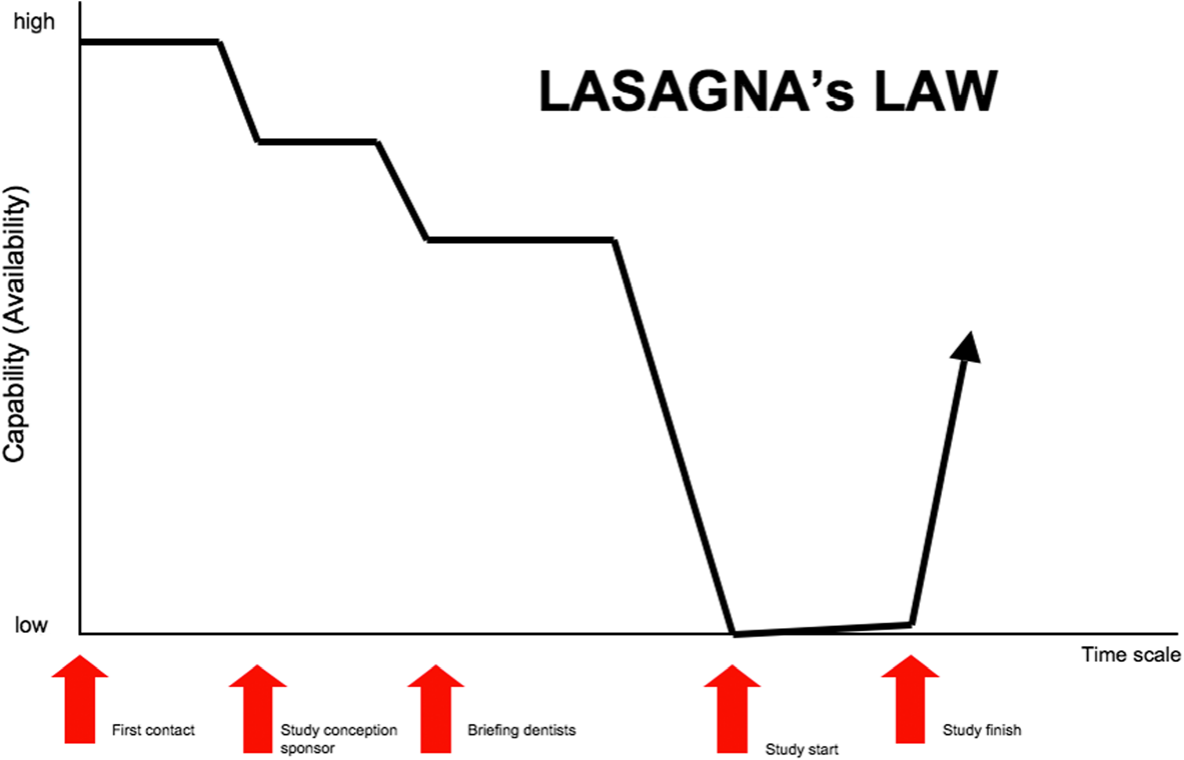
The number of participants dropped to low levels the day the trial started and increasing the number of participants required alternative planning

Nonetheless, we faced delays in the recruitment period because fewer dental clinics than expected actually participated in the lectures and gave their final confirmation. This overestimation at the early stages of recruitment, or what can be described as a discrepancy between planning and reality, is explained by a phenomenon known as Lasagna's Law [22] (Fig. 3), where researchers overestimate the number of participants in a clinical trial. Therefore, a large discrepancy is found between practitioners who initially agree to take part in the study and practitioners who start recruiting patients immediately [23–27]. Huibers et al. [28] acknowledged some of the factors that are thought to influence this phenomenon, such as unmotivated practitioners or practitioners who are not able to recruit eligible patients, whereas Gray et al. [29] emphasized that time constraints and team motivation are the most important factors to obtain higher participation rates. Furthermore, in a review of 41 clinical trials, Charlson et al. [30] reported that only 24 trials reached approximately 75 % of their proposed number of recruited patients, and they concluded that longer recruitment periods have to be considered for a clinical trial to reach its targeted number of recruited patients. McDonald et al. [31] reviewed 114 clinical trials and found that the original recruitment target was achieved in less than a third (31 %) of the trials; they noted the complexity and high costs of prolonging the recruitment process and that other factors that must be considered, such as rates of participant retention and treatment compliance.

Although we found that we overestimated the willingness of participation, the final rate of participation was comparable to other clinical studies in the field [4, 32–34], and a response of 4.5 % from the overall invited dental clinics would still yield the required representative sample size of patients and subsequent fillings. Furthermore, the literature shows that participation rates in clinical studies differ largely according to the study settings and design. Moreover, the participation rate in clinical studies requiring actual physical examination or treatment tends to be lower in comparison to survey-only studies [9, 35, 36].

We tried to find a relation between the socioeconomic status of each region's population and recruitment rate and how these factors influence the willingness of participation. We initially looked at the household income and the state of unemployment as previsions on the participation rate; however, we found that using only the rate of unemployment or household income cannot be used to predict the participation rate, but rather the combination of household income or rate of unemployment and the population density (inhabitants per km^2^) showed a significant relation. We noticed that more practitioners had participated in regions with low income or high rates of unemployment and low population density (Regions 2 and 3) (Table 2).

We also noticed that the practitioners who participated in this clinical trial were either specialized dental practitioners who showed an interest in scientific research (31 % were specialized in periodontology, 20 % in pediatric dentistry, 9 % in oral surgery, and 9 % in orthodontics) or young practitioners who were open minded and more educated about clinical trials than their older peers.

## Conclusion

Limited information and knowledge are available on what conditions or factors influence the recruitment of dental practitioners in clinical trials. Regional differences in socioeconomic status, practitioner specialization, and patient health care insurance need to be considered while recruiting. Furthermore, the recruited practitioners must be trained and calibrated according to the international standards in clinical trials (CONSORT Criteria, GCP).

The results from the dental practitioner recruitment in this study suggest that the recruitment and pre-randomization design were successful, and by reaching out to a considerable number of private dental clinics to participate, we were able to recruit a smaller number of highly motivated dentists who were willing to incorporate their patients in this clinical study.

## Additional files

Additional file 1:
**Trial Protocol – English.** (DOCX 5363 kb) Additional file 2:
**Trial Protocol – German.** (PDF 197 kb) Additional file 3:
**SPIRIT checklist.** (DOC 228 kb) Additional file 4:
**CONSORT checklist.** (DOC 324 kb) Additional file 5:
**CONSORT flow diagram.** (DOC 51 kb)

## Abbreviations

ART: atraumatic restorative treatment
DSMC: Data Safety and Monitoring Committee
GCP: good clinical practice
GIC: glass ionomer cement
GMP: good manufacturing practice
RCT: randomized clinical trial
SCS: single-center studies
SES: socioeconomic status
