# Supplementary material for: Dental practitioner recruitment for a randomized clinical trial in the field to evaluate the performance of a new glass ionomer restoration material
Source: Trials. 2016 Feb 10;17:73. doi: 10.1186/s13063-016-1198-3 (PMC4748549; doi:10.1186/s13063-016-1198-3)
Supplement: Additional file 1: — Trial Protocol – English. (DOCX 5363 kb) [file 13063_2016_1198_MOESM1_ESM.docx]

**Study-Operating-Procedure (SOP) of**

**„Clinical Success of the**

**filling alternative „EQUIA^®^“**

**(Fa. GC Europe N.V., Belgien)“**

Dr. Th. U. Klinke

Prof. Dr. R. Biffar

Ernst-Moritz-Arndt-University

Center of Oral Health

Piloclilnics of Prosthodontics, Gerostomatology

and Dental Materials

(Chair: Prof. Dr. R. Biffar)

Rotgerberstrasse 8

Walther-Rathenau-Str. 42a

D-17489 Greifswald

Version 2_engl

# Background of the project?

The benefits of glasionomer fillings were shown in literature. Nevertheless, in Germany glasionomer fillings are not accepted to be permanent restorations for the adult population. The new developed material EQUIA® (*Fuji IX GP Extra* coated with *G-Coat Plus®* ) gives –regarding manufacturers expectations- a new opportunity because of a combination of adhesive linkage to the teeth, esthetic improvements and special coating. Other benefits are x-ray opacity, biocompatibility, low sensitivity to moisture, low shrinkage. The main benefit of the new material –regarding manufacturer’s expectations- is the increase of mechanical strength. A nano-filled coating (GC G-Coat Plus) protects the surface initially from erosion. The product is introduced under the rules of MPG into the German market and is CE certified.

# Aims of the Study

The aim of this prospective clinical trail as cohort study according to MPG phase IV is ethical proved at the Ethical Comitee of Greifswald University (BB/2009) registerd at the WHO (DRKS-ID: **DRKS00004220**)

- To compare the survival rate of EQUIA^®^ (*Fuji IX GP Extra* coated with *G-Coat Plus^®^)* further named as “**Equia**” on the one hand and GIC *Fuji IX GP^®^ fast* coated with *Fuji Coat LC^®^* further called as “**Fuji IX**” on the other hand as filling material in posterior teeth.
- To compare the wear characteristics of EQUIA^®^ and Fuji IX
- To evaluate the renew ability of EQUIA^®^ and Fuji IX

Hypothesis is that the survival rate of EQUIA^®^ will be better than Fuji IX.

# Head of project

Prof. Dr. R. Biffar

OA Dr. Th. Klinke

Greifswald University

Center of Oral Health

Walther-Rathenau-Strasse 42a

17475 Greifswald

Tel.: +49-3834-867140

FAX: +49-3834-867148

[Biffar@uni-greifswald.de](mailto:Biffar@uni-greifswald.de)

[Klinke@uni-greifswald.de](mailto:Klinke@uni-greifswald.de)

# Examination

OA Dr. Th. Klinke

Greifswald University

Center of Oral Health

Walther-Rathenau-Strasse 42a

17475 Greifswald

Tel.: +49-3834-867140

FAX: +49-3834-867148

klinke@uni-greifswald.de

# Dental Treatment

Dentists in participating dental offices were randomly recruited regarding SOP. They

confirmed participation regarding SOP. Recruitment procedure takes place in

randomly selected cities with more than 50 thousands and less than 250 thousand

inhabitants regarding SOP.

Contact during study:

OA Dr. Th. Klinke and N.N.

# Statistics and Data Safety

Dr. dipl. math. Chr. Schwahn

Greifswald University

Center of Oral Health

Walther-Rathenau-Strasse 42a

17475 Greifswald

Tel.: +49-3834-867140

FAX: +49-3834-867148

[schwahn@uni-greifswald.de](mailto:schwahn@uni-greifswald.de)

## Data Safety and Monitoring Comitée (DSMC)

Prof. Dr. R. Hickel

Dep. of Restorative Dentistry

LMU Munich

Goethestr. 70

80336 München

Tel. 089-5160-9301

Fax 089-5160-9302

[hickel@dent.med.uni-muenchen.de](mailto:hickel@dent.med.uni-muenchen.de)

# Sponsor

GC Europe N.V.

Interleuvenlaan 33

3001 Leuven, Belgium

Tel :  +32 16 74 51 32

Cell : +32 479 84 48 24

Fax : +32 16 74 51 40

Contact person: Dr. Piyush Kandelwal (Be),

GC Germany GmbH

Seifgrundstraße 2

61348 Bad Homburg

Deutschland

Tel: +49 6172 99596-0

Fax: +49 6172 99596-66

Contact person: Frank Rosenbaum (D), Monika Schwandner (D)

# Study Type

Double Blinded – randomized – prospective – investor – initiated clinical trail

# Duration

5 years after the recruiting of participating dentists in their own dental practice

(proposed period of recruiting: 0.5 y, proposed period of treatment of participants:

0,5 y). Yearly Follow-up: start point: first placed filling).

# Calculation of the participants

The calculation of the numbers of participants were done with „nQuery 4.0“ software

by Dr. Dipl-Math Chr. Schwahn.

As aim (dependent variables) were used

- Loss of filling (dichotomy variable), defined as fracture or crack of the filling
- Abrasion of the occluded filled surfaces (scaled variable)
- Wear abrasion of the occlusal surface (scaled variable)

When the sample size in each group (Fuji IX and Equia) is 440, an exponential

maximum likelihood test of equality of survival curves with a p= 0.050 two-sided

significance level will have 90% power to detect the difference between a Group 1

exponential parameter, .1 of 20 % 0.0201 (corresponding to a proportion of 30% after

60 months) and a Group 2 exponential parameter, .2 of 0.0268 (corresponding to a

proportion of 20% after 60 months and a constant hazard ratio of

0.0201/0.0268=0.75) 30 %. This assumes an accrual period of 12 months, a

maximum follow-up time of 60 months (five years), and a common exponential

dropout rate of 1%.

The calculation is based on all incorporated units (fillings), e.g. all possible units

(fillings). The homogeneity of the participants will be guaranteed by the recruiting

phase, which will use the following selection criteria: Only one (at most two)

restoration for each side is calculated for each patient. The restoration will be limited

to posterior teeth (premolars and/or molars).

# Data Safety and Monitoring Committee

The cases will be pseudonymized (continuously counted according to a single main

list). The Data-Safety-and-Monitoring-Committee (DSMC) will guarantee the

abidance of randomization and quality assurance at the data acquisition and data

base. Every six months the DSMC will forward a half-year report. Prof. Dr. Reinhard

Hickel was asked for serving as a DSMC member and gave a preliminary consent. A

second member of DSMC will be one of the members of our board of the community

medicine research net with expertise in statistical methodology.

# Pseudonyms

Each dental practice gets a pseudonym (ID-OFFICE), which is known by only one

member of the research/examiner team in Greifswald (keyholder). Opening of the

pseudonym can only be done by DSMC and the keyholder in a formal consent.

Subjects included in the cohort will get a pseudonym (ID-PROBAND) by the

participating dentist. The dentists are the keyholders for the patients personal data.

The list of pseudonyms and patients´ ID will remain in the dental office. Only the

dentists (keyholder) could open the pseudonyms in their (private office) dental office.

The pseudonyms of ID-OFFICE and ID-PROBAND will allow complete data

calculation regarding the aims of the study.

# Recruitments

The recruitments of the dentists will be carried out in a two-stage design. Dentists will

be selected in a randomized design before the clinical trial will start.

The main project will start definitely after finishing the recruitments of dentists and

evaluation by the DSMC. In the dental practices patients will be recruited for the

study by a randomized list.

## Recruitment of dentists (1st Stage)

German cities of 50-250T inhabitants will be identified and 8 randomly selected.

The dentists will prepare the tooth and place the filling in the selected dental practice

regarding given manufactures recommendations; Dental practices will be randomly

selected in cities of more then 50 to 250T inhabitants. A short proposal will be sent to

30 randomly selected dental offices in each of the cities. Mailing to further dental

offices will be continued till 10 dentist per city confirmed participation.

Participating dentists will sign a short contract form and will be educated in the

sufficient application of GIC according to the manufacturers’ instruction and

according to GMP by GC including a training about the needed procedures written in

the SOP regarding the special research methods used in this study.

## Recruitment of patients (2nd Stage)

The patients are visitors of the cooperating dental practice, where the indication of

fillings in posterior teeth is indicated. A starting date will be given to the dentist, to

define when to start recruitment of patient by a randomized list. All participants, who

are randomly selected, will sign a consent form. If a patient is not willing to

participate, he/she will be asked along a short form of questionnaire (3 Items:

education, dental attendance, last visit of a dentist). Afterwards the list switches over

to the next random number.

The calculation of the fillings (see point 7) per participant (dentist and patients) in

different cities can be estimated by the mean of 1 to 3 fillings per patient (Mean 2)

and 3 to 8 patients (mean 5.5) per office, which were treated by 8 dentist in 10 cities.

In the study can be included 240 fillings at minimum, in the max 1920 fillings can be

calculated. If the number per dentist will not reach estimated mean, further dentists

will be recruited by the randomized design.

|  | filling/patient | patients/dentist | dentists/city | cities | expected | recommended |
| --- | --- | --- | --- | --- | --- | --- |
| **min** | **1** | **3** | **10** | **8** | **240** | **440 each arm** |
| **max** | **3** | **8** | **10** | **8** | **1920** |  |
| **mean** | **2** | **5,5** | **10** | **8** | **880** |  |

This calculation requires a continuously calculation of the included fillings in the study, to give a contemporary feedback to the participating dentist.

Additional cohorts (such as “GC opinion leaders”) are not included in this randomized

controlled study (RCS), but they can be added as “additional selected” cohorts

(ASC). They will be calculated separately and used as additional information for

discussion.

# Participants

Dentate patients attending the dental office, where the indication of a filling (amalgam

or composite) in the posterior region is given and who signed the consent form /

study contract (appendix).

## Inclusion criteria

Dentate patients (no partial or full dentures, proximately 3 opposing zones with

contact of natural teeth in the posterior region)

Fillings are limited to two surfaces (o, mo, od) but with one proximal cavity. The width of the cavity should not exceed 50% of the tooth distance and the approximal cavity width is limited to the cusp tip distance.

## Exclusion criteria

Patients with CMD dysfunction or malfunction (grating or articular crepitus, pressing)

will be excluded from the study. The entire screening method (CMD-Screening

according to DGZMK record) has been evaluated in previous research projects of the

study group (SHIP communication).

Participants, who may not be willing to sign the consent form, will be withdrawn to

take part in the clinical investigation (according to Declaration of Helsinki and

Guidelines ICH-GCP). Three questions will be ask for non-responder analysis and

sex and birthyear from patients data file will be noted instead of participation.

# Clinical Trial

Before patients will be included in this clinical trail, they must sign the consent form

and the acceptance of randomization. A Xerox of the patient consent form will be

given to the patient (participant).

The main list of randomization decides the treatment strategy and is oblique to be

included in the study. Patients which do not agreed with the randomization will be

excluded of the participance. The reason for withdrawal will be recorded (Question:

Which expectance did you established to the restoration material?)

According to indication, the cavity will be grained and an impression of the cavity

(Optosil) be undertaken. Ten questions regarding age, education, dental attendance,

oral hygiene, smoking etc. will be asked for the study record. The questions are part

of SHIP instrument (Study of Health in Pomerania, University of Greifswald).

The study box, handed out after the briefing lecture, contains blinded, numbered envelops, which contains only one single capsule. The use of this randomized material is binding. The capsule differs optically in the colour of the stamp. The colour code of the stamp shows, which coating (G-Coat LC or G-Coat Plus) has to be used in the last step. Is the necessity of an additional filling at the same volunteer indicated, the other study material will be used.

The filling will be placed according to manufacturers´ recommendations. To finish the

filling procedure, the surface has to be coated with G-Coat LC or G-Coat Plus (which

was used out of blinded, relabelled bottles by GC, Belgium) according to the random

list. Manufacturers´ recommendations and research methods will be trained before

(see above).

Regarding pseudonym procedure the patient will be registered by the next number in

the list and the envelope of randomization list will be opened to define the therapy

arm (G-Coat LC or G-Coat Plus). The bottles will be relabelled by “coat number A or

B”. The random list will tell the dentist to use bottle A or B. Only the project

management could identify the coat by the given numbers. So, neither the dentist nor

the participant will know in this blinded design which coat material was used.

*The materials get blinded and randomized by GC Europe. Only the information of the*

*expired date, colour (yellow, grey) is given to the participants.*

After cure of the material, the shape of the filling has to get contoured (diamant 10-20 µm) and sealed according to the manufactured recommendation.

Conserving the shape of the filling for the study a replica will be done by one phase A-silicone (EXA fast Injection, GC Company) and impression tray with a setting time of prox. 360 sec. The impression methods will be trained during education of filling procedures. Interocclusal records (interponates) will be done with Green-bite apple (Co: Detax, Germany) during baseline and the follow-ups in central occlusion.

The participant gets a unique pseudonym in the dental office. The unique pseudonym

remains in the list of the dental office.

The filling protocol/record with the randomized envelope number, Probands ID, setting date, treated tooth/teeth and surface has to be filled out. The impression gets named with the randomized envelope number and Probands ID. After all fillings are set, the filling record has to be send to the University Greifswald.

# Examination

A member of the project team (calibrated examiner) will evaluate the fillings after the

first, second and the fifth year after placement. Criteria (see 16.1) out off the

publication of Hickel, R. et al. “Recommendations for conducting controlled clinical

Studies of dental restorative materials” (Clin Oral Invest (2007) 11: 5-33) and Hickel et al_ FDI World Dental Federation: clinical criteria for the evaluation of direct and indirect restorations – update and clinica lexamples. Clin Oral Invest (2010) 14: 349–366, Appendix) will be used for examination.

The examiners will be calibrated once a year by external examiners. Additional the tool e-calib (www.e-calib.info) has to be used by the examiners for training purposes. The project team will organize the recall of the follow up in the local dental office in collaboration with the dentists.

To reduce costs the participating dentists will evaluate by their own for the baseline,

after the third and forth year. Replicas will be sent to the project team for evaluation.

## Criteria

The criteria of failure are published in Hickel, R. et al., Clin Oral Invest (2007) 11: 13; Update Clin Oral Investig 2010 14(4), 349-66 and Hickel et al_ FDI World Dental Federation: clinical criteria for the evaluation of direct and indirect restorations – update and clinica lexamples. Clin Oral Invest (2010) 14:349–366. Criteria A1, B5, B6, B7, B8, B10, C11, C12 and C13 will be included in the study (See Appenix).

| A1: Surface lustre |
| --- |
| B5: Fracture of material and retention |
| B6: Marginal adaptation |
| B7: Occlusal contour and wear |
| B8: Approximal anatomical form |
| B10: Patient´s view |
| C11: Postoperative sensitivity and tooth vitality |
| C12: Recurrence of caries, erosion, abfraction |
| C13: Tooth integrity |

If the patient will attend between the examination dates, failures will be counted with their first occurrence, documented in patients’ record and study protocol.

For Follow-up explorers (Deppeler, Suisse) will be used: Dental explorer 150 µm and 250 µm, matrix band 50 µm, 100 µm and 150 µm thickness.

## Examination after application of the filling

The data files (Screening, questionnaire) and baseline impression will be sent by mail

to the project team for analysis by scanning. The data files create a data base (Stata

10.1). All impressions will be scanned (transmission and surface scan). Transmission

scans gets analysed with GEDAS tool *(Hützen D, Proff P, Gedrange T, Biffar R, Bernhard O, Kocher T, Kordass B: Occlusal contact patterns – population-based data. Ann Anat. 2007;189(4): 407-11; Hützen D, Rebau M, Kordass B: Clinical reproducibility of GEDAS – „Greifswald Digital Analysing System“ for displaying occlusal patterns. Int J Comput Dent 2006 Apr;9(2):137-142. )*

The measurements include the isthmus thickness in extend and depth of the occlusal

surface to the mesial or distal preparation by di- and trihedral fillings. For evaluation

greater fillings (more then trihedal) only the box preparation can be counted.

Randomly selected replica of case- and control arm will be scanned (3DLaserscan/

SmartOptics, accuracy < 20 µm).

## Follow Up

A Follow up is planned after the first to the fifth year after application. If the filling

failed (see 13.1 Criteria) and has to be replaced, no further follow up will be

necessary.

To reduce costs for examination baseline, third and fourth follow up examination will

be executed by the dentist.

The first, second and fifth follow up examination will be hold by external, calibrated

examiner (dentist), who does intraoral photographs, replica of the shape of the filling

using EXA fast Injection (GC Company) and interponate (Green-bite). Further examination like dental appraisal will not be recorded.


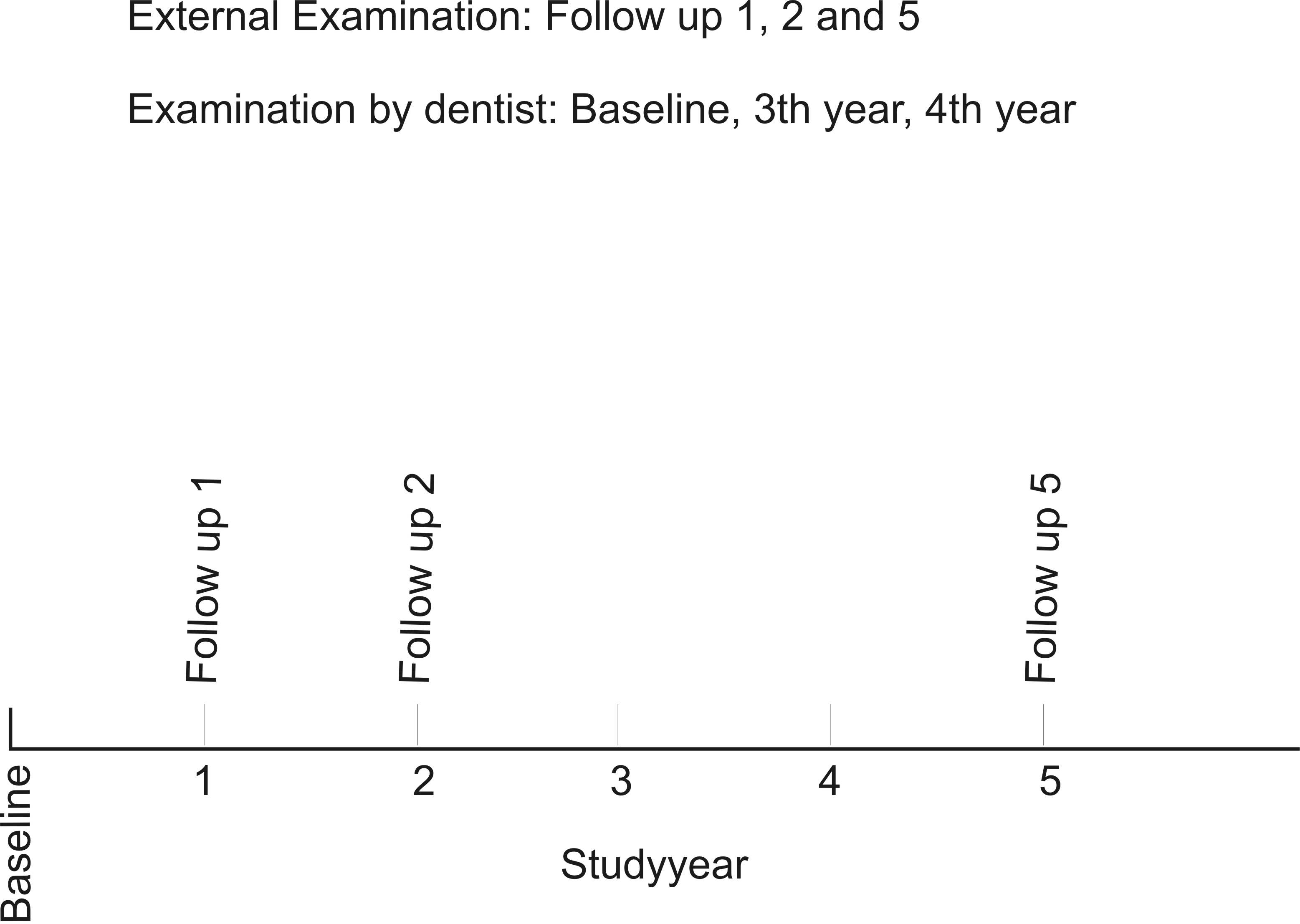


# Interpretation strategy

The adverse event (“Failure”, according to Hickel, R. et al.) in the study- and control

arm will be calculated in Kaplan/Meier and/or Hazard ratio.

The size of the teeth contact situation in transmission scans will be explored with

GEDAS. The representative randomized selected scan data of the replica gets

matched with the Baseline (Software-Matching) to calculate the volume loss and

height loss in wear characteristics (continuos variables).

Statistical calculation will include descriptive statistics, linear logistic regression,

multivariate logistic regression, Hazard and Kaplan Meyer estimation.

In the case of failure, the patients’ record contains date of failure or date of renewing.

Study relevant clinical record files (CRFs) will be collected at the Greifswald

University and archived for 15 years after study closure.

For the archive contains:

- - Participant signed consent form
  - Insertion RF
  - Follow up RF
  - Occlusal impressions/Interponates
  - Pictures and fotographs
  - Statistical data file and output

# Cut off points

The study will be suspended if recruitment of dentists will not reach the estimated

numbers per cities during 6-9 month.

The study will be suspended, if failure rate reaches more then 5% of the fillings in the

first year.

The study will be suspended, if response will be below 60% of the examination each

year in respect to the year before.

Not used materials will be sent back to GC.

# Monitoring

A monitoring in the local dental offices will be arranged, of 10% of 880 fillings are

placed. The external examiner will check the sufficiency of study documents

(probands signed consent form, Insertion RF e.g.). Queries will get debugged.

# Study report

Each year the sponsor obtained a preliminary report of the study results in the first

second, third and forth study year. A final report will follow after the fifth year. Regular

Newsletters inform the participating dentists of the results and intermittent failures.

# Responsibilities

- LKP: Prof. Dr. Reiner Biffar

OA Dr. Thomas Klinke

- PI: OA Dr. Thomas Klinke and calibrated examiner
  - Application for ethical board
  - Assiting statistical evaluation of data (done by Dr. Dipl. math. Chr. Schwahn)
  - Recruitment of local dental offices
  - Calibration of participating dentists
  - Contact person of participating dentists
  - SOPs (Study-Operating-Procedure)
  - Information form for participants
  - Consent form
  - Steering of baseline and Follow Ups 3,4
  - Follow Ups 1,2,5
  - Study files, documentation and archives
  - Randomization
  - Organisation of study groups per city
  - Records and communications
  - Final record
  - Publications
  - Cost control
- Examiner:
  - External Examiner (University Greifswald, University Marburg)
    - Local scheduling
    - Examiantion during Follow-up
    - Files, documentation
    - Report of adverse events to project group
    - Local data archive
    - Participation in study group
  - Dentist of local dental office
    - Files, documentation
    - Recruitment of participants
    - Local scheduling
    - Report of adverse events to project group
    - Local data archive
    - Participation in study group
- Statistics: project team (Dr. Dipl.-Math. Chr. Schwahn)
  - Data Management
  - Data Safety
  - Statistical reports
  - Statistical calculations
- Medical documentalist
  - First Data entry (double entry N.N.)
  - Data management
  - Control of CRF
  - Feed back of Qeuries
- Data Safety and Monitoring Committtee (Prof. Dr. Hickel and N.N.)
  - Data Management Control
  - Data Safety Control
  - Data Quality Control
  - Randomization Procedure Control
  - Veto: If quality reaches not the estimated level.
- Sponsor

o Event analysis

o Audit of files and archives

o Resources and financial support

o Organisation an arrangements of study groups and initial training

## Study File

- Permission of ethical board
- Consent forms of participants
- Insurance policy
- Documentation of treatment procedure
- Anamnestic data
- Documentation of examination parameters
  - - Local dental office:
      - Application of restoration, baseline examination
      - Date and data of examinations
      - Invitations Follow Up 1,2,3,4,5 in cooperation with project team
      - Examinations of Follow Ups 3,4 and baseline
      - Impressions regarding SOP of Follow Ups 3,4 and Baseline
    - Calibrated examiner:
      - Date and Data of examinations
      - Examinations Follow Up 1,2,5
      - Impressions regarding SOP of Follow Ups 1,2,5
      - Data file of scanning processes

## Organisation of Follow Up

- Organisation of Follow Up during study period of 5 years
- Organisation and scheduling of examinations (local dental office and
- project team)

## Communications

- Communications with project team during Follow Ups (local dental office)
- Schedule appointments in cooperation with project team
- Rejected appointments
- Communications, if adverse events occur (local dental office)
- Communication with project team
- Communications of project team
- Communications with sponsor
- Communications with local dental office

## Recruitment of participants

- Local dental office
- Control of randomization procedure by project team and DSMC

## Randomization

- Randomized controlled clinical trial
- Control of randomization by project team and DSMC

## Meeting of study groups

- project team and GC
- Organisation, invitation, training and support by GC

# Appendix

## Ethical approval

## Trail registration

This trial was approved by the ethical commision at Greifswald University (No: BB33/09) and ist registered at WHO (DRKS:-ID:DRKS00004220) at <http://drks-neu.uniklinik-freiburg.de/drks_web/setLocale_EN.do>


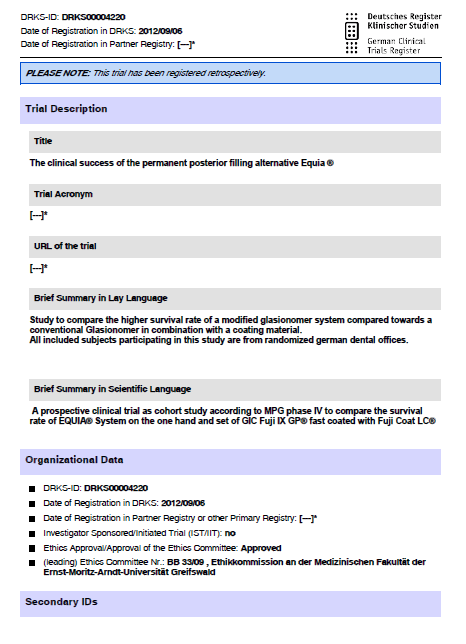


##
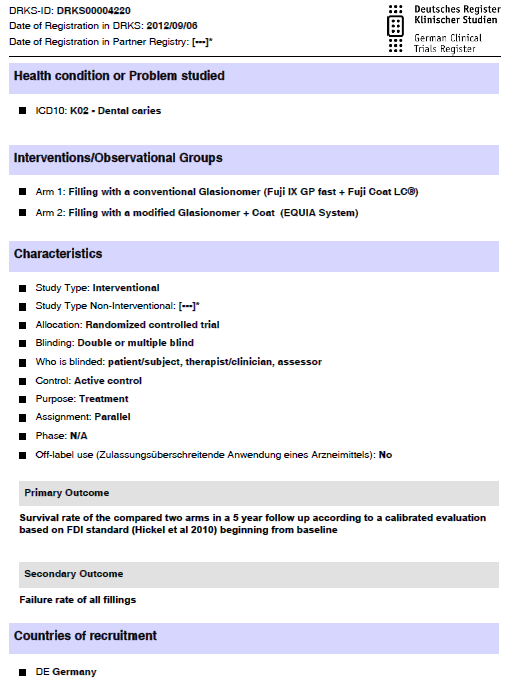


##
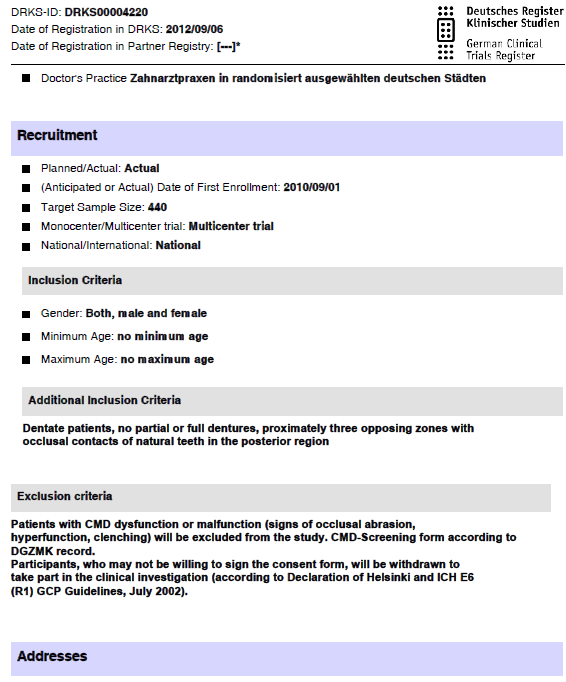


##
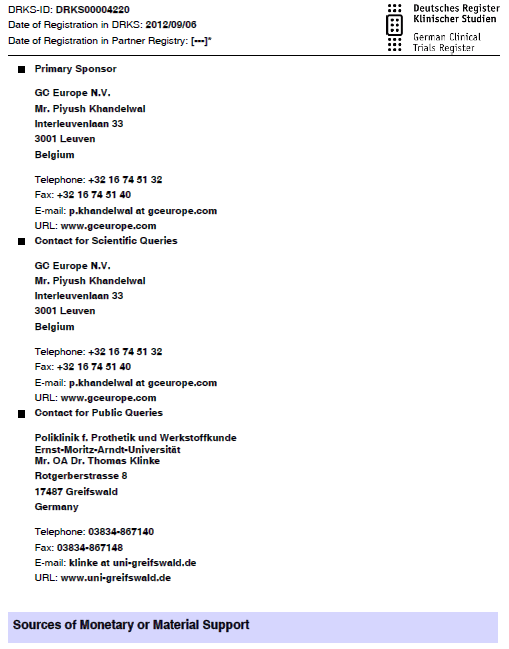


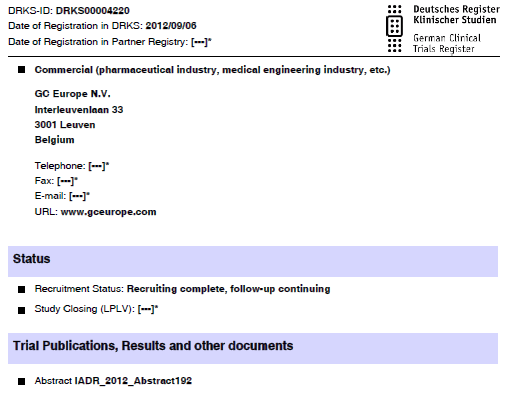


## Recruitment 1^st^ step: Numbers and areas

* incl. expansionof 25 km

## Questionnaire for the participating dentist Pseudonym: 🞏🞏🞏🞏

Location of practice: __________________________________________

**Where is your office located:**🞏 city center
🞏 city outskirts
🞏 surrounding rural areas of the city

What year was your final exam? 19 🞏🞏

For how many years is your practice? 🞏🞏

**Which practice areas do you have?**🞏 General Dentistry
🞏 Periodontology
🞏 pediatric dentistry
🞏 Orthodontics
🞏 Oral Surgery
🞏 Oral and Maxillofacial Surgery
🞏 Other _______________________

**How many dentists treat, except you, in the practice?**1 2 3 4
🞏 🞏 🞏 🞏 assistant dentist / doctors / inside
🞏 🞏 🞏 🞏 partner / inside (clinic)
🞏 🞏 🞏 🞏 partner / inside (practice sharing)
 🞏 no

**How many patients do you treat about a year?**
Health insurance (number of patients): approx ______

Private patients (invoice number): approx ______

What is the proportion of refarral patients: ____ %

Do you have a recall system?
           🞏 yes 🞏 no

How many patients engage your recall system?: ____ %

## Questionnaire for the participating patients:

Pseudonym : 🞏🞏🞏🞏

Age: 🞏🞏 Gender 🞏Female 🞏 Male

Marital Status:

🞏 married

🞏 verheiratet / separated

🞏 currently live in a committed relationship

🞏 been single, never married

🞏 divorced

🞏 widowed

How many years in schooling you have successfully completed ?

🞏 up to 8 years

🞏 8 to 10 years

🞏 more than 10 years

What is your highest degree?

🞏 still a student ( in ) without completion
🞏 Volks- or Hauptschule
🞏 Leaving school without final

🞏 High school, technical school degree, Fachschulreife

🞏 Degree of the polytechnical school

🞏 College degree, technical university, technical school
🞏 High School, general university
🞏 College degree, skilled workers with high school
🞏 other graduation

What do you think about the health of your teeth?

🞏 very good

🞏 well

🞏 satisfactory

🞏 less well

🞏 bad

How are your teeth cleaning habits?

🞏 once a week

🞏 once a day

🞏 twice a day

🞏 three times or more a day

Do you use futher oral hygiene products beside your toothbrush?

🞏 yes

🞏 no

How often were you in the last 10 years the annual bonus check ?

🞏🞏 times

When was the last time before the dentist?

🞏 this year

🞏 last year

🞏 two to three years ago

🞏 more than four years ago

## Proband’s information and consent (Translation from german)

**Proband’s information and consent
to conduct a clinical investigation of a medical product**

**Study Center**: University Greifswald, Dental School, Department of Prosthodontics,

Gerodontology and Material Science

**Investigator:** OA Dr. Thomas Klinke, Prof. Dr. Reiner Biffar

Greifswald University, Department of Prosthodontics, Gerodontology and Materials Science
Rotgerberstrasse 8, D-17487 Greifswald, Phone (+49)3834-867 140

**Clinical performance of the filling alternative EQUIA**

Dear Madam, Dear Sir,

we ask, whether you would be willing, to participate with your planned filling restorations in a **clinical controled trial for long-term behavior**. Your dentist is one out of 10 cooperative dentists in your city. In the routine examination, done by your dentist, a **carious leason was detected in the posterior region, which has to be treated with a dental filling to stop further destruction of the tooth**. Due to the inclusion criteria, your dentist has the opportunity to include you as a proband in the clinical trial described here.

**Clinical trials in dentistry are needed in order to increase or expand the knowledge about the durability and resistance of fillings.** This clinical trial, which we introduce here, was assessed by the relevant **ethics committee approval** - as the law requires -. This clinical trial is being conducted in eight cities in Germany; it will include a total number of about 220 patients. The study was initiated and is conducted by the University of Greifswald. It is funded by the dental material manufacturer GC Europe NV, Interleuvenlaan 33, B-3001 Leuven, Belgium and GC Germany GmbH, Seifgrundstraße 2, D-61348 Bad Homburg, Germany, as the sponsor of this clicnical trial.

**Your participation in this clinical trial is voluntary**. So you will only be included in this study, **if you sign this proband’s acquiescence**. **If you do not wish to participate in the clinical study or retire later from her, no disadvantages arise for you.**

Your dentist has already given you a range of information about the proposed study. The following text should give you a closer explanation of the aims and procedure. Subsequently, an informed consent discussion is carried with you. Please do not hesitate to ask all the points which are unclear. You will then get a reasonable period of time to decide your participation.

**1. Why is this study done?**

**GC Fuji IX GP Extra and fast, GC G-Coat Plus and LC are approved dental filling material for this purpose in Europe and are used in the routine treatment after the removal of tooth decay as tooth filling material for a long time and been assessed as equivalent filling material.** They are strong withstanding the chewing pressure and keep the teeth free of caries. The fillings have to fulfil two essential tasks in addition to the repair of the defective site in the teeth: They must meet your aesthetic requirements and needs to withstand the forces of chewing. Unfortunately there is not a material that meets all demands on time. The wear and fracture of the fillings are known and recurring events.

**In the planned clinical trial will be studied over a period of five years, whether there are differences in the wear behavior and fracture behavior between these two products of the same manufacturer. The two products belong to the same class of materials (glass ionomer cement) and differ only slightly in the composition of ingredients and their processing.** The study described here **is not a material testing of a new material**, but the reliability of known materials over the duration of the study is examined here.

**2. Do I get one of the investigational dental products anyway?**

**In the context of this clinical study, two previously assessed as equivalent materials are used.** If you participate, you will receive either GC or GC Fuji IX GP Equia. **Which filling material can be used, decides a previously defined random process, comparable to the toss of a coin; this is called randomization. The probability to obtain one out of the two materials, is 50%.**

For an objective data collection it is necessary that neither you nor your dentist know which material is selected (this process is called a "double blind"). Should it be necessary for safety reasons, it can be determined immediately which material you have received. The mapping is stored in a list at the University of Greifswald.

**3. What is the process of the study and what should I consider for participation?**

Before inclusion in this clinical study, you will asked about your marital status, years at school, highest final degree, your personal estimation of your dental health, your tooth brushing habits and your last dental visit.

If you participate in the study, you are required to attend a check-up per year over a period of five years. You also can combine this investigation with the usual annual dental check-up bonus booklet according with your dentist. For the follow-up after one, two and five years, an examiner of the University of Greifswald will examine the fillings in the dental office. He only will examine the fillings, which were placed in the context of this study, to detect chipping, fractures and breakage. In addition to that, he will take two small dental impressions with silicone impression material. The dental impressions document the changes to the fillings. The investigation of the remaining teeth is performed by your dentist and is not associated with this study. For the follow-up in the upcoming years, an appointment for the examination of the fillings will be agreed by you and your dental practice and you will be invited to attend it.

Please understand, that only these appointments will be done, in which all participants of the study can take part. It would help us greatly, if you could enable the proposed appointments, because an external examiner from the university Greifswald far arriving extra for this follow-up. The examinations after three and four years of follow-up are led by your dentist. In the case that a filling which is under observation, is damaged, we ask you, if at all possible, seek out as soon as possible the practice of your dentist. If you are unable to visit you dentist, please share your dentist without delay, that a filling has become defective. Because this dental filling material is approved for this purpose, this sustained damage is a general risk, that even without participation in the study, could occur.

**4. What personal advantage do I have from study participation?**

You do not have any personal advantage by study participation, neither any personal health benefits. However, the results of the study about dental fillings and their longevity will improve the treatment in the future.

**5. Which are the risks associated with participating in the clinical trial?**

From participating in the study, no other or even increased risk arises in comparison to other dental filling materials this product category. Specific side effects are not known for these products.

**6. Who may not participate in this clinical trial?**

You can not participate in this clinical trial, if you grind and press or suffer permanent muscle problems of the masticatory muscles, if you wear a removable partial or complete dentures and have less than three successive teeth biting.

**7. Do I incur costs by participating in the clinical trial? Do I get an allowance?**

By participating in this clinical trial no incur cost to you. For regular participation in the study over five years you will receive a grant in the amount of 70 EUR for professional dental cleaning at your dentist.

**8. Will I be covered by insurance in the clinical trial?**

We point out, that you are insured on the way to the dental office for the examination.

**Name and address of insurance:**

(adress written in the german Patient’s consent)

**9. Will I get informed about new findings clinical examination disclosed?**

You will be informed by your dentist about new findings that are known with respect to this clinical trial and that may be essential for your willingness to participate further. On this basis, you can then rethink your decision to further participation in this clinical trial.

**10. Can I terminate my participation in the clinical trials ahead of schedule?**
You can terminate your participation at any time, without giving any reason. You will not incur any drawbacks. But under certain circumstances it is also possible that the study center or the sponsor decides to prematurely terminate your participation in the clinical trial ahead of schedule, without that you have influence on the decision. The reasons for this can for example the early termination of the entire clinical trial.

**11. What happens to my clinical data?**

During the clinical trial all medical findings and personal information will be collected in your personal file in the dental office or stored electronically. **Very important: For the clinical trial data is additionally stored in only a pseudonym, evaluated and passed by the dentist at the study center of the University of Greifswald. Pseudonym means that no details of names or initials are used , but only a number and / or letter code , if necessary, maybe the year of birth. The key to the code remains in the dental practice you can trust and is managed by your dentist.**

All collected data are protected against unauthorized access. Decryption is performed only under the conditions prescribed by law. Data to the yearly check-ups are processed only in the dental practice you can trust. If you do not agree to the use of your data in the form described, your participation in the clinical trial is impossible.

**Details, in particular on the possibility of a cancellation, please refer to the consent form, which are printed at the final chapter on this proband's information and consent form.**

**12. What happens to my impressions?**

Your dental impressions are used exclusively for this clinical study. You will be electronically recorded and stored under the respective pseudonym.

**13. Whom do I contact for further information?**

Counseling sessions at the study center. You always have the opportunity to further consultations with the investigator listed on page 1 or another. Please feel free to contact him.

**Study Center**: University Greifswald, Department of Prosthodontics, Gerodontology and Medical Materials

**Investigator:** OA Dr. Thomas Klinke, Prof. Dr. Reiner Biffar

Department of Prosthodontics, Gerodontology and Dental Materials Science
Rotgerberstrasse 8, D-17487 Greifswald, Phone 03834-867 140

**Clinical performance of the filling alternative EQUIA**

**Declaration of consent**

............................................................................................................................
Name of the subject in block letters


birthday: ................................ Probands number:..........................................


I am in a personal interview by the participating dentist


............................................................................................................................
Name of dentist

been elucidated fully and clearly about the clinical trial as well as the nature, significance, implications and risks of the clinical trial. I also have read and understood the text of informed and here reprinted below privacy policy. I had the opportunity to speak with the investigator about the conduct of the trial. All my questions were answered satisfactorily.
 
Way of documenting additional questions from the subjects or other aspects of the education conversation:

I had plenty of time to decide.

I am aware, that I can withdraw my consent to participate in the examination at any time without notice for any reason (oral or written) without giving me any incurred that it disadvantages.

**Privacy Policy:**

I am aware that to be in this clinical study, personal data, particularly medical records collected, stored and analyzed by me. The use of the information regarding my health is based on statutory provisions and sets before participating in the clinical trial following voluntarily given informed consent ahead, that is, without the subsequent agreement can not participate in the clinical trial.

1 I hereby agree that in the context of this clinical study, personal data, in particular information about my health, about me collected and recorded on paper and on electronic media with my dentist. The data collected are pseudonymous (encrypted) are passed to the testing center in Greifswald:

a) to the study center, the sponsor or a person, authorized by the latter for the purpose of

Scientific evaluation,

c ) in the case of adverse events: at the trial site, the sponsor, to the appropriate Ethics Committee and the competent higher federal authority *Federal Institute for Drugs and Medical Devices (Bundesinstitut für Arzneimittel und Medizinprodukte)* and from this to the European database.

2 In addition, I give my consent that authorized and obliged to secrecy officer of the sponsor and the authorities responsible for monitoring in my existing dentist personal data, especially my health information , insight, so far as is necessary for the verification of the proper conduct of the study. For this measure, I release the dentist and the principal investigators of the test center of medical confidentiality.

3 The consent to the collection and processing of my personal data, in particular the details of my health is irrevocable. I was already informed that I may at any time terminate participation in the clinical trial. In the case of such a withdrawal of my consent to participate in the study, I hereby agree that the stored up to that point data may be used further to obtain the sufficient power of the study.

4 I hereby agree that my personal data will be retained after the completion or termination of the study at least ten years as it determine the rules concerning the clinical investigation of medical devices. After that my personal data will be deleted, unless there are statutory and statutory retention periods.

5 I am aware of the following legislation: If I express my consent to participate in the study revoke, all the bodies that have my personal data, especially health data stored, immediately check the extent to which the stored data for the in # 3 purposes referred to are still required.

No longer needed data must be deleted immediately.

**With my signiture, I am willing**

**to participate as an voluntare this clinical trial mentioned above.**

I received a copy of the proband’s information and consent. One copy remains in the study center Greifswald of Department of Prosthodontics, Gerodontology and Dental materials.

...........................................................................................................................
Name of the subject in block letters


............................................ .................................................................
Date Patient’s Signature

I informed and discussed with the patient and obtained the consent of the patient.

...........................................................................................................................
Name of dentist

.................................................. ................................................................
Date Patient’s Signature

## Placement Protocol (Translation from german)

**Treatment Protocol Study Center Greifswald**

**FAX 03834-86 7148**

After the placemt of the filling, please insert the Proband’s ID, the date of birth as well as the insertion date, the treated tooth / teeth and surfaces in the clinical record file.

| Envelope | Proband’s ID | Date of birth | Insertion date | Treated tooth/teeth |
| --- | --- | --- | --- | --- |
| ***Example*** | *# 3456* | *1.2.1965* | *21.6.2009* | *15mo, 16od* |
| **1** |  |  |  |  |
| **2** |  |  |  |  |
| **3** |  |  |  |  |
| **4** |  |  |  |  |
| **5** |  |  |  |  |
| **6** |  |  |  |  |
| **7** |  |  |  |  |
| **8** |  |  |  |  |

Dental office ID

## Clinical Record File (CRF, Translation from German)

## Clinical Record File (CRF) Follow Up NU __     ____

| **Dental office ID _****______________** | |
| --- | --- |
| Date of Follow up: |  |
| Patient’s ID: | Pseudonym: |
| No:      Interponat: Replica: | |

| Treatment | 1 | 2 | 3 | 4 |
| --- | --- | --- | --- | --- |
| Tooth |  |  |  |  |
| Surface (S1, S2, S2mod) |  |  |  |  |
| A1  Surface lustre |  |  |  |  |
| B5  Fracture of material and retention |  |  |  |  |
| B6  Marginal adaptation |  |  |  |  |
| B7  Occlusal contour and wear |  |  |  |  |
| B8  Approximal anatomical form |  |  |  |  |
| B10  Patient’s view |  |  |  |  |
| C11  Postoperative (hyper-)sensitivity and tooth vitality |  |  |  |  |
| C12  Recurrence of caries (CAR), erosion, abfraction |  |  |  |  |
| C13  Tooth integrity |  |  |  |  |

See: Hickel et al

# Evaluation Criteria

See: Hickel et al_ FDI World Dental Federation: clinical criteria for the evaluation of direct and indirect restorations – update and clinica lexamples. Clin Oral Invest (2010) 14:349–366

#
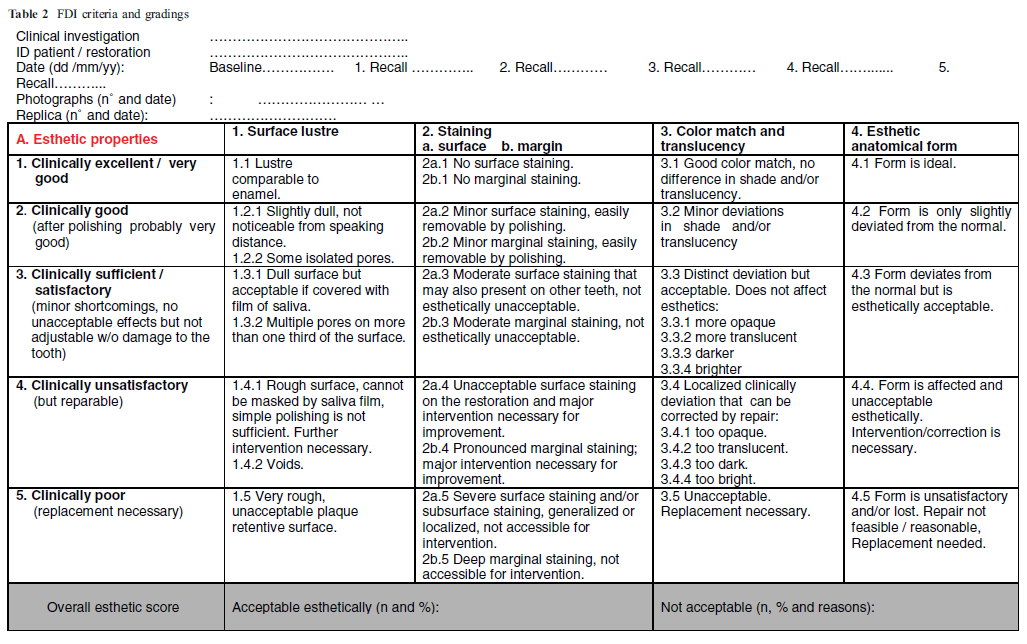


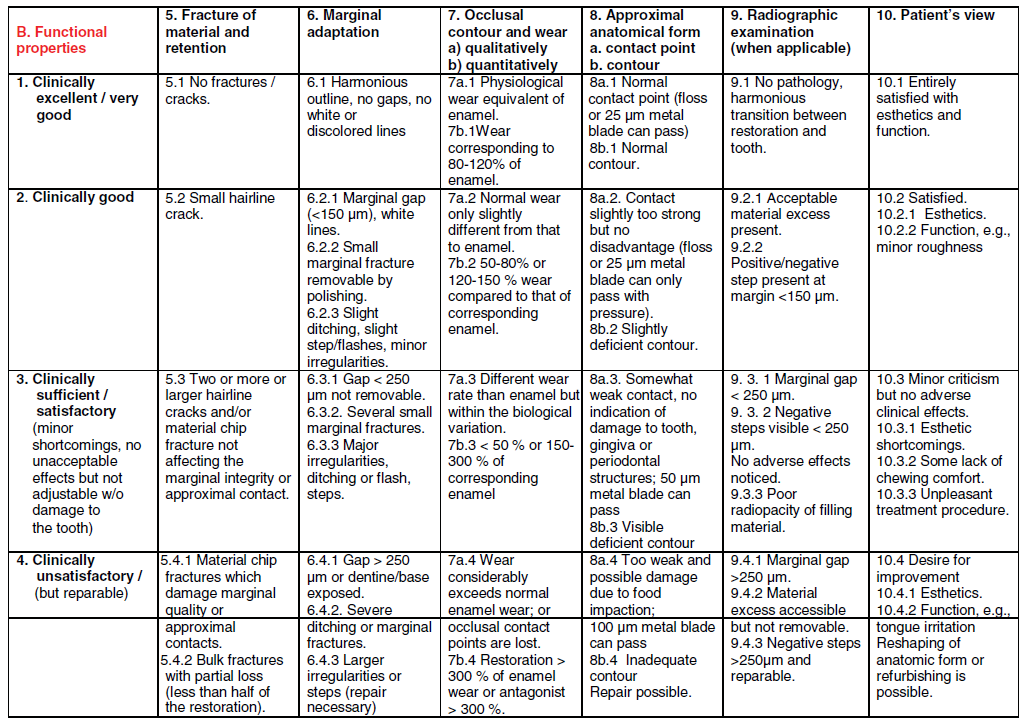


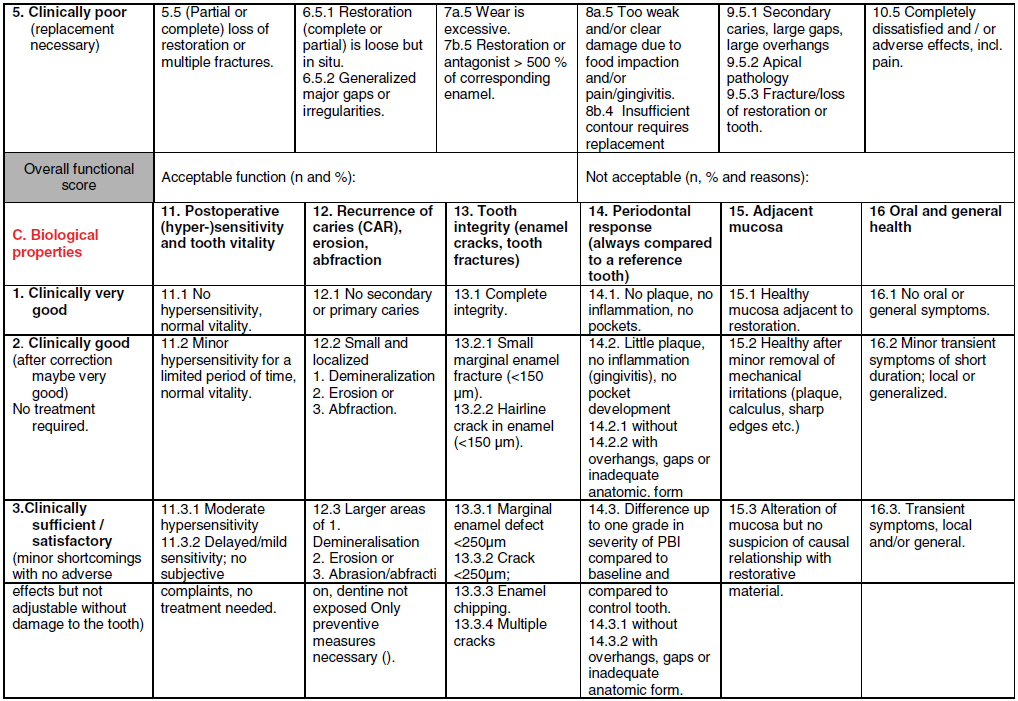


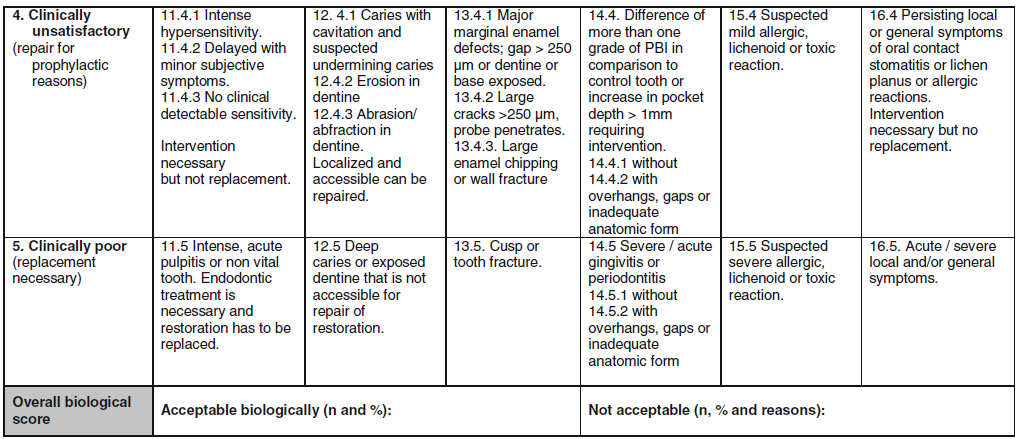


# Notes
