# Supplementary material for: Dental practitioner recruitment for a randomized clinical trial in the field to evaluate the performance of a new glass ionomer restoration material
Source: Trials. 2016 Feb 10;17:73. doi: 10.1186/s13063-016-1198-3 (PMC4748549; doi:10.1186/s13063-016-1198-3)
Supplement: Additional file 2: — Trial Protocol – German. (PDF 197 kb) [file 13063_2016_1198_MOESM2_ESM.pdf]

Handlungsplan
(Study-Operating-Procedure)

Klinische Bewährung der definitiven Seitenzahnfüllungsalternative Equia®
(Fa. GC Europe N.V., Belgien)

Vorgelegt von

OA Dr. med. dent. Th. Klinke
Ernst-Moritz-Arndt-Universität
Poliklinik für zä. Prothetik, Alterszahnheilkunde und med. Werkstoffkunde
Dir.: Prof. Dr. R. Biffar
Rotgerberstrasse 8
17475 Greifswald

Inhaltsverzeichnis

Klinische Bewährung der definitiven Seitenzahnfüllungsalternative Equia® 1
1 Einleitung 4
1.1 Einsatzbereiche.. 4
1.2 Mechanische Festigkeit 4
1.3 Methodik der Applikation 6
1.4 Bioverträglichkeit... 6
2 Studienziel 7
3 Projektleitung 7
4 Klinischer Prüfer 7
5 Zahnmedizinische Arbeiten... 8
6 Datensicherung und –auswertung 8
7 Sponsor 8
8 Probanden 9
9 Studientyp.. 9
10 Dauer der Studie 9
11 Kalkulation der Probandenzahl 9
12 Rekrutierung der Zahnarztpraxen.. 10
12.1 Rekrutierung und Behandlung der Patienten 10
13 Vorgehensweise 11
13.1 Untersuchung nach Füllungsapplikation... 11
13.2 Follow Up.. 12
13.3 Auswertungsstrategie 12
14 Abbruchkriterien 13
15 Monitoring... 13
16 Data-Safety-Monitoring-Comitée 13
17 Berichterstattung 13
18 Verantwortlichkeiten und Zuständigkeiten 14
18.1 Prüforder... 14
18.2 Studienplan abgestimmt mit Standard... 15
18.3 Therapieplan abgestimmt mit Standard.. 15
18.4 Jährliches Follow up organisieren 15
18.5 Info aller Beteiligten.. 16
18.6 Rekrutierung der Patienten.. 16
18.7 Randomisierung 16
18.8 Dokumentation 17
18.9 Studientreffen 17
19 Literatur... 18
20 Anhang 20
20.1 Patientenaufklärung... 21
20.2 Einverständniserklärung.. 22
20.3 Beispiel für Füllungsprotokoll 31
20.4 Beispiel für Nachuntersuchungsbogen.. 32

1 Einleitung

Seit mehr als drei Jahrzehnten werden Glasionomer-Zemente in den Zahnarztpraxen verwendet. Basierend aus einem Gemisch aus Carbonsäuren (z. B. Polyacrylsäure oder deren Copolymere), anorganischen Füllkörpern (Kalzium-Aluminium-Silikat-Glas) und destilliertem Wasser, härten Glasionomer-Zemente durch eine Säure-Basen-Reaktion aus. Das Füllungsmaterial, das überwiegend in unterschiedlichen Farbtönen zahnfarben verfügbar ist, wird hauptsächlich bei Kindern für Milchzahnfüllungen und bei bleibenden Zähnen für provisorische Füllungen, z.B. während einer Schwangerschaft, verwendet.
Aufgrund der Haftungsmechanismen am Zahnschmelz als auch am Dentin macht ihn für die Zahnmedizin besonders attraktiv.

1.1 Einsatzbereiche

Indikation	Kontraindikation
• Milchzahnfüllungen (eingeschränkt)<br>• Fissurenversiegelung (eingeschränkt)<br>• Zahnhalsfüllungen<br>• provisorische Füllung<br>• Unterfüllung<br>• Aufbaumaterial für Kronen und Brücken<br>• Befestigungszement für Kronen/Brücken<br>• Befestigungszement für kieferorthopädische Bänder	• endgültige Füllung für Kauflächen im Seitenzahnbereich des bleibenden Gebisses

Im Vergleich zu einer Amalgamfüllung ist die Lebensdauer zwar reduziert (Chadwick et al. 2002), dafür enthält aber Glasionomerzement Fluorid, das kontinuierlich an den Zahn abgegeben wird. Durch diese Fluoridabgabe - die kurz nach dem Legen der Füllung am größten ist - an die Füllungsumgebung wird ein automatischer Kariesschutz im Füllungsbereich bewirkt. Deshalb sind Glasionomer-Zement-Füllungen vor allem im kindlichen Gebiss eine gute Wahl.

1.2 Mechanische Festigkeit

Zur Steigerung der Materialeigenschaften, z. B. die Biege- und Abriebfestigkeit der herkömmlichen Glasionomer-Zemente zu verbessern, wurden in den 80er-Jahren des vorigen Jahrhunderts Versuche mit Metallzusätzen (z. B. Silber) vorgenommen. Unter hohen Temperaturen wurden die Metallteilchen an das Glaspulver gebunden. Diese

metallverstärkten Glasionomerzemente bezeichnet man als Cermet-Zemente (Ceramik-Metall-Glasionomer-Zemente). Die erhoffte Verbesserung der Materialeigenschaften konnte in verschiedenen Untersuchungen nicht bestätigt werden (Frankenberger, R., Krämer, N.: Glasionomerzemente). Da es bei diesen Zementen aufgrund ihres Metallgehaltes zu Korrosion und Verfärbungen des Zahnes kommt, sind diese Modifikationen als Füllungs- und Aufbaumaterial nicht geeignet.

Die größte Stabilität kann bei modifizierten Glasionomerzementen erreicht werden, wenn sie in Verbindung mit kompositbasierten Adhäsivsystemen eingesetzt werden, so venezolanische und US-amerikanische Wissenschaftler in einer neuen Publikation (Setien, V J et al, 2005). Resin-modifizierte Glasionomerzemente bieten gegenüber konventionellen Glasionomerzementen (GIZ) nicht nur verbesserte mechanische Eigenschaften, sondern zeichnen sich auch durch einfache Verarbeitung aus. Forscher aus Venezuela und USA untersuchten jetzt die Bruchfestigkeit modifizierter GIZ bei Verwendung verschiedener Konditionierungstechniken in einer werkstoffkundlichen Studie (Setien, V J et al, 2005).

Aus menschlichen extrahierten Zähnen wurden standardisierte Probekörper hergestellt. Die Dentinflächen wurden mit Hilfe eines der folgenden Verfahren konditioniert:
1.) 20% Polyacrylsäure, 3% Aluminiumchlorid (Cavity Conditioner®, GC);
2.) 35% Phosphorsäure, Primer (Scotchbond-Multipurpose System®, 3M ESPE);
3.) 35% Phosphorsäure, Primer, Adhäsiv (Scotchbond-Multipurpose System®, 3M ESPE).
Anschließend wurde Restaurationsmaterial (Fuji LC®, GC) eingebracht und lichtgehärtet. Nach Lagerung der Proben für 1 bis 24 Wochen in künstlichem Speichel oder Öl wurde maschinell die Bruchfestigkeit bestimmt.

Insgesamt wiesen die in Technik 3 verarbeiteten Proben die höchste Bruchfestigkeit auf. Zudem konnte eine signifikante Abnahme der Stabilität im Testzeitraum von 1 bis 24 Wochen festgestellt werden. Die Lagerung in Öl wirkte sich aufgrund des verminderten Feuchtigkeitseintritts in allen Gruppen positiv auf die Stabilität aus. Die Autoren empfehlen aufgrund ihrer Ergebnisse die Verwendung von Adhäsivsystemen bei direkten Restaurationen mit modifizierten GIZ.

Anstelle von Metallen können auch Kunststoffe zugesetzt werden. Diese kunstoffmodifizierte Glasionomer-Zemente werden durch Licht (z. B. Halogen- oder LED-Licht) ausgehärtet.

Eine dänische Studie, in der über die Dauer von acht Jahren das Schicksal von 500 Amalgam und Glasionomer-Zement-Füllungen bei Kindern im Alter von 3 bis 13 Jahren untersucht wurde, konnte den Kariesschutz der Glasionomer-Zement-Füllungen bestätigen. Bei Amalgamfüllungen mussten die Nachbarzähne der gefüllten Zähne, die im direkten Kontakt zur Füllungsmasse standen, in 30% aller Fälle wegen Karies behandelt werden. Bei Glasionomer-Zement-Füllungen waren es lediglich 16%. Es sollte allerdings nicht verschwiegen werden, dass nach einer Literaturauswertung durch Nathanson (2000) der Kariesschutz nicht schlüssig bewiesen ist. Aufgrund der Karieshemmung durch Fluoridabgabe und der relativ einfachen Verarbeitung der Glasionomerzemente werden sie derzeit erfolgreich im Rahmen der von der WHO geförderten ART-Versorgung ("Atraumatic Restoration Treatment" = Füllungsbehandlung ohne Bohrer) in Entwicklungsländern verwendet.

Bei der ART-Technik wird Karies mit Hilfe von Handinstrumenten entfernt und anschließend der Zahn mit Glasinonomerzementen gefüllt. Eine Anästhesie wird nicht benötigt, da durch

den Verzicht auf den schnelllaufenden Bohrer in der Regel kein Schmerz entsteht. Diese Art der Behandlung ist für ländliche Gebiete in Entwicklungsländern entwickelt worden, in denen kein elektrischer Strom zum Betrieb zahnärztlicher Instrumente zur Verfügung steht. Der Bevölkerung soll mit dieser einfachen Behandlung zumindest ein Mindestmaß an zahnmedizinischer Versorgung ermöglicht werden. Das es funktioniert, zeigt eine chinesische Studie (Hu et al.) aus dem Jahr 2004: kleine einflächige ART-Füllungen waren nach 6 Jahren noch zu 75% und große einflächige noch zu 61% vorhanden.

Das neue Material EQUIA® bestehend aus GC Fuji GP Extra® und G-Coat PLUS® stellt lt. Herstellerangaben einen neuen Ansatz in der Füllungstherapie dar. Auf Basis von GIZ (GV Fuji IX GP EXTRA) wurde dieses Füllungsmaterial entwickelt, das die Vorteile der Verankerungsmöglichkeit von GIZ (chemischer Verbund zu Dentin und Schmelz) einerseits und die ästhetischen Eigenschaften von Composites (optische Eigenschaften durch speziell entwickelte Glaskörper) in sich vereinigen. Weitere Vorteile sind Röntgenopazität, Bioverträglichkeit, geringe Feuchtigkeitssensitivität (keine absolute Trockenlegung notwendig), kein Schrumpfungsrisiko. Bezüglich der mechanischen Eigenschaften ist die Härte oberhalb der Festigkeit von Compositewerkstoffe. Durch den lt. Herstellerangaben verwendeten Schutzlack auf Grundlage eines nanogefüllten Schutzlackes (GC G-Coat Plus) wird die Oberfläche initial vor einem Auswaschungseffekt durch Wasserzutritt geschützt, später durch Infiltration die Verschleißfestigkeit erhöht.

1.3 Methodik der Applikation

Zunächst wird der erkrankte Bereich entfernt (Füllungsdefinierung, Exkavation) und die Kavitätenränder geglättet. Eine Unterfüllung (Zinkoxid-Phosphatzement) gewährleistet bei tiefen Defekten eine Abdeckung der Dentintubuli. Zur verbesserten Adhäsion an Dentin wird ein Liner appliziert und Glasionomer-Zement im Mischer nach Aktivierung gemischt. Das plastische Material wird anschließend in die Kavität appliziert. Damit das Material ungestört vom Auswaschungseffekt (Speichelzutritt) aushärten kann, wird die Füllung mit einem Schutzlack abgedeckt.

1.4 Bioverträglichkeit

Berichte über Allergien auf *Glasionomer-Zemente ohne Kunststoffzusatz* ("konventionelle Glasionomerzemente") liegen nicht vor. *Glasionomer-Zemente mit Kunststoffzusatz* enthalten Monomere (z. B. 2-Hydroxyethylmethacrylat (HEMA)), die als Allergene bekannt sind. Es gibt jedoch bisher nur wenige Berichte über Patienten mit allergischen Reaktionen auf Acrylate in Zahnfüllungen. Als Symptome werden Stomatitis, Mundbrennen, Ekzeme im Mundbereich und Utikaria genannt (Arenholt-Bindsley, Kanerva 2005). Unter bestimmten Umständen ist auch eine Formaldehyd-Freisetzung möglich.

2 Studienziel

Mit dieser prospektiven, epidemiologischen Kohorten-Studie (Phase IV, MPG) soll im Untersuchungszeitraum der Nachweis erbracht werden,

- dass der Glasionomerzement (GIZ) Fuji IX GP Extra mit G-Coat Plus, im weiteren als ***„EQUIA***®“ bezeichnet, im Seitenzahnbereich eine höhere Überlebenswahrscheinlichkeit aufweist, als der gängigen GIZ Fuji IX GP fast in Kombination mit Fuji Coat LC im folgenden als ***„Fuji IX“*** bezeichnet,

- dass die Abrasionseffekte („Wearcharacteristics“) von Equia (insbesondere mit der Vergleichsgruppe : Fuji IX) verbessert ist,

- dass die Erneuerungswürdigkeit von Equia oberhalb von der mittleren Lebensdauer von Fuji IX liegt.

3 Projektleitung

Prof. Dr. R. Biffar
OA Dr. Th. Klinke
Poliklinik für Prothetik und Werkstoffkunde
Ernst-Moritz-Arndt-Universität
Rotgerberstrasse 8
17487 Greifswald
Tel.: 03834-867140
Fax: 03834-867148
biffar@uni-greifswald.de,
klinke@uni-greifswald.de

4 Klinischer Prüfer

OA Dr. Th. Klinke
Poliklinik f. Prothetik und Werkstoffkunde
Ernst-Moritz-Arndt-Universität
Rotgerberstrasse 8
17487 Greifswald
Tel.: 03834-867140
Fax: 03834-867148
klinke@uni-greifswald.de

5 Zahnmedizinische Arbeiten

Kollegen in Kooperationspraxen, die randomisiert ausgewählt wurden und die Studienteilnahme signalisiert haben. Die Rekrutierung der Kollegen (5 – 7 Kollegen) erfolgt durch Anschreiben der Praxen in Städten (> 50 – 250 T Einwohner).

Ansprechpartner:
n.n.

6 Datensicherung und –auswertung

Dr. dipl. math. Chr. Schwahn
Poliklinik f. Prothetik und Werkstoffkunde
Ernst-Moritz-Arndt-Universität
Rotgerberstrasse 8
17475 Greifswald
Tel.: 03834-867140
Fax: 03834-867148
chr.schwahn@uni-greifswald.de

Datenauswertung und –sicherung für die Dauer von 15 Jahren nach Studienende

7 Sponsor

GC Europe N.V.
Interleuvenlaan 33
3001 Leuven, Belgium
Tel : +32 16 74 51 32
Cell : +32 479 84 48 24
Fax : +32 16 74 51 40

Ansprechpartner: Khandelwal, Piyush [p.khandelwal@gceurope.com]

GC Germany GmbH
Seifgrundstraße 2
D-61348 Bad Homburg
Tel.: 06172/99 596-0
Fax: 06172/99 596-66

Ansprechpartner: Frank Rosenbaum, Monika Schwandner

8 Probanden

Bezahnte Patienten der Praxen, bei denen die Indikation der Füllungstherapie mit Amalgam bzw. mit Seitenzahnkomposites besteht, die ihr Einverständnis zur Studie gemäß Einverständnisständniserklärung (s. Anhang) erklärt haben.

Einschlusskriterien:
Normal bezahnt (kein abnehmbarer Zahnersatz, mindestens 3 Stützzonen)

Ausschlusskriterien:
Anzeichen für kraniomandibuläre Dysfunktionen – gemäß funktionellem Screening-Befund – Zielkriterien sind Befunde zu Knirschen und Pressen.

Patienten, bei denen die Einverständniserklärung nicht vorliegt, werden nicht in die Studie aufgenommen. Es werden den Kriterien nach GCP entsprochen (siehe *Deklaration von Helsinki, Guidelines ICH-GCP*)

9 Studientyp

Blinded – randomized – prospective - clinical trail

10 Dauer der Studie

5 Jahre ab Rekrutierung der beteiligten Zahnärzte in Niederlassung (hiervon 1 Jahr Rekrutierung und Versorgung von Patienten). Follow Up-Untersuchungen in jährlichem Abstand geplant.

11 Kalkulation der Probandenzahl

Die Kalkulation des Stichprobenumfanges wurde mittels des Programms „nQuery 4.0“ von Herrn Dr. dipl.math. Chr. Schwahn durchgeführt.

Als Zielereignisse werden
- Verlust der Füllung (dichotome Variable), definiert als Bruch der Füllung.
- Abrasion in kontakttragenden Arealen (kontinuierliche Variable)
- Abrasion der Okklusionsflächen (kontinuierliche Variable)

formuliert.

Das Programm ermittelte, dass jede Gruppe (Fuji IX und Equia) 440 Einheiten umfassen soll. So beträgt die Power 90% (Signifikanz p= 0,05) dafür, dass nach 60 Monaten (bzw. 5 Jahren) ein Unterschied zwischen zwei Gruppen nachgewiesen wird, wenn die eine Gruppe einen Anteil an verbliebenen Restaurationen von 30% und die andere von 20% aufweist. Dabei wird

ein Rekrutierungszeitraum von bis zu 24 Monaten, eine maximale Beobachtungsdauer von 60 Monaten und eine (exponentielle) Drop-Out-Rate von 1% angenommen.

Die Kalkulation bezieht sich auf eingegliederte Einheiten, darunter fallen alle Restaurationen. Für die Rekrutierung werden zur Gewährleistung der Homogenität des Probandengutes folgende Einschlussbegrenzung eingeführt: Pro Patient können in je einer Kieferhälfte nur eine Restauration gewertet werden, maximal jedoch zwei Einheiten pro Proband. Die Versorgungen werden auf den Seitenzahnbereich (Prämolaren/Molaren) beschränkt.

Die jeweiligen Fallzahlen werden fortlaufend registriert. Die Einhaltung der Randomisierung und die Qualitätssicherung der Datenerhebung werden durch ein Data-Safety-and-Monitoring-Committee (DSMC) überwacht. Das DSMC wird halbjährlich einen Bericht des Projektteams erhalten. Das DSMC wird aus zwei Mitarbeitern der Medizinischen Fakultät bestehen (ein Biomathematiker, ein wissenschaftlicher Assistent (Arzt) aus dem Bereich Community Medicine). Jeder Praxis wird ein Praxiskennzeichen (Praxispseudonym) zugeordnet. Die Liste ist nur einem Mitarbeiter des Projektteams zugängig, über den/die alle Kontakte zu den beteiligten Zahnärzten geleitet werden. Außer diesem Mitarbeiter sind allen anderen Mitarbeitern im Projektteam die Namen und Adressen der Zahnärzte nicht bekannt. Die Verarbeitung der Daten ist ohne Offenlegung der Identitäten über die Pseudonyme von Zahnarzt und Patienten möglich.

12 Rekrutierung der Zahnarztpraxen

In der Rekrutierungsphase erfolgt in einem zweistufigem Design, in der die teilnehmenden Kollegen (ca. 300 Praxen) angeschrieben und zur Teilnahme eingeladen. Sollte die Rekrutierung nicht erfolgreich sein, werden weitere 200 Praxen angeschrieben. Als Einzugsgebiete werden randomisiert 10 Städte mit 100.000 bis 250.000 Einwohnern deutschlandweit ermittelt. Aus jeder Stadt werden randomisiert 30 Praxen angeschrieben mit dem Ziel je 8 Praxen nach Eingangsdatum einschließen zu können. Wird die Zahl von 8 Praxen nicht im ersten Durchgang erreicht, wird anhand der randomisierten Liste der weiter angeschrieben und eingeladen.
Von den teilnehmenden Praxen (80 Praxen) sollen je Füllungsmaterial 12 Füllungen bei unterschiedlichen Patienten gelegt werden.

Die Kalkulation der Anzahl der Praxen und Patientenanzahl pro Therapiearm in dem 440 Einheiten eingeschlossen werden, können wie folgt kalkuliert:

	Füllung/Patient	Patient/Zahnarzt	Zahnarzt/Stadt	Stadt	Ist	Soll
Min	1	3	8	10	240	**2x440**
Max	3	8	8	10	1920	
Mittelwert	2	5,5	8	10	**880**	

12.1 Rekrutierung und Behandlung der Patienten

Die Rekrutierung erfolgt nach der Rekrutierungsliste, die vor Behandlungsbeginn vorhanden ist. Darin wird festgehalten, ob der Patient auf die Studie hingewiesen wird, wenn eine Indikation zur Versorgung besteht. Im Aufklärungsgespräch werden den Patienten Zweck, Inhalt, Durchführung, Dauer und Risiko der Studie erklärt. Der Patient gibt sein Einverständnis zur pseudonymisierten Verwendung seiner Daten und erlaubt dem Projektteam und seinem Zahnarzt ihn/sie zu weiteren Follow Up Terminen anzuschreiben und einzuladen. Die spezielle Patienten-/Probandeninformation wird dem Patienten ausgehändigt.

13 Vorgehensweise

Das Einverständnis des Patienten (auch über die Akzeptanz der Randomisierung) wird eingeholt. Eine Kopie der Einverständniserklärung wird dem Patienten ausgehändigt.
Das Siegel der randomisierte Liste wird geöffnet und die Therapiealternative festgelegt und ist bindend für die Aufnahme in die Studie. Patienten, die mit dem Randomisierungsergebnis nachträglich nicht einverstanden sind, werden ausgeschlossen und der Grund für diese Entscheidung als Klartext notiert. (Frage: Was waren Ihre Erwartungen, die Sie an Ihre Wahl des Therapiemittels geknüpft haben?)
Entsprechend der Indikation (ein- oder zweiflächige Füllung im Seitenzahnbereich) werden die Kavität nach zahnärztlichen Belangen beschliffen, die Kavität mit einem Durchbissregistrat abgeformt (knetbares kondensationsvernetzendes Silikon (zB. Optosil)) die Füllung(en) wird nach den Vorgaben der Hersteller gelegt. Nach der Applikation der Füllung wird nach Aushärtung und Konturierung der Füllung wird die Füllung mit einem Lack gegen Feuchtigkeitszutritt gesichert. Die Entnahmeflaschen sind verblindet (etikettiert als Flasche A oder Flasche B) und beinhaltet entweder Fuji Coat LC oder G-Coat Plus. Die Auswahl welche Flasche verwendet wird, ist der zweiten Randomisierungsliste zu entnehmen und zu dokumentieren. Nach Füllungslegung und Überprüfung der Okklusion wird die Oberfläche mit einem Interponat (z.B. Greenbite/Fa. Detax) abgeformt und eine Replik aus Futura Scan (Fa. Kettenbach) hergestellt (Baseline).
Jedem Probanden wird in der jeweiligen Praxis ein Pseudonym zugeteilt, dass in der praxiseigenen Liste geführt wird. Außerhalb der Praxis können die Daten nur über das Pseudonym zugeordnet werden.

13.1 Untersuchung nach Füllungsapplikation

Das Registrat und der Untersuchungsbogen (Screnning und Basisfragen zum Patienten) werden unter den Pseudonym-Kennzeichen von Patient und Arzt an das Projektteam per Post gesandt. Die Daten aus Untersuchungsbögen werden in die Datenbank aufgenommen Alle Interponate werden im Durchlichtscan und Auflichtscan erfasst. (GEDAS*) Aus den Interponaten wird der Isthmus der okklusalen Fläche zum mesialen bzw. distalen Kasten bei zwei- und dreiflächigen Füllungen gemessen. Bei mehr als dreiflächige Füllungen kann nur bei Kastenpräparationen gemessen werden.
Repliken von einer randomisiert gezogenen Untergruppe aus Fall- und Kontrollarm werden eingescannt (3D-Laserscan/ SmartOptics, Genauigkeit < 20 µm). (siehe *Befund*).

13.2 Follow Up

Follow Up ist geplant nach 1, 2, 3, 4 und 5 Jahren nach Applikation. Ist die Füllung schadhaft und muss ausgetauscht werden, ist das Zielereignis eingetreten und weitere Follow Up werden ausgesetzt.
Das ‚Follow Up 3' und ‚Follow Up 4' werden vom beteiligten Zahnarzt durchgeführt. Das ‚Follow Up 1', ‚Follow Up 2' und ‚Follow up 5' werden durch einen externen Prüfzahnarzt (Uni Greifswald) durchgeführt.
Bei allen Follow Up-Terminen werden die Restaurationen auf Frakturen und Defekte untersucht und ein Interponat gefertigt.
Zu dem Follow Up 2 und 4 wird vom Prüfarzt zusätzlich eine Fotodokumentation intraoral und jeweils eine zusätzliche Replika aus Futura Scan (Kettenbach) angefertigt (siehe Anhang *Nachuntersuchungsbogen*). Eine weiterreichende Befundung führt der Prüfarzt nicht durch.

13.3 Auswertungsstrategie

Zielereignisse „Verluste" in Fall- und Kontrollarm werden in Kaplan-Mayer- und/oder Hazard-Schätzungen dargestellt.

Die Durchlichtscans werden bezüglich der Kontaktbeziehungen auf Pixelebene ausgewertet (GEDAS*, Hützen et al.). Hieraus kann der Verlust im Kontaktbereich der Antagonisten errechnet werden.
Die gescannten Daten aus Repliken werden anschließend gematcht (Software-Matching), der Volumenverlust, Höhenverlust wird zur Baseline im Zweijahres-Rhythmus errechnet.

Im Schadensfalle dokumentiert der Defektbogen den Zeitpunkt des Verlustes/Erneuerungszeitpunkt. Die Unterlagen werden abschließend an der Universität Greifswald gesammelt und der Archivierung zugeführt. Dauer der Archivierung 15 Jahre nach Studienabschluss.

(*)
1. **Hützen D, Proff P, Gedrange T, Biffar R, Bernhard O, Kocher T, Kordass B:** Occlusal contact patterns – population-based data. Ann Anat. 2007;189(4): 407-11
2. **Hützen D, Rebau M, Kordass B:** Clinical reproducibility of GEDAS – „Greifswald Digital Analysing System" for displaying occlusa patterns. Int J Comput Dent 2006 Apr;9(2):137-142

Zur Archivierung gelangen:

- Einverständniserklärung
- Eingliederungsbogen
- Nachuntersuchungsbogen ggf. Defektbogen (ZA; Uni, Fa. GC)
- Modelle/Daten der okklusalen Abformung
- Fotografien
- Auswertung der wissenschaftlichen Daten

14 Abbruchkriterien

Die Studie wird beendet, wenn das Studienziel nicht erreicht wird und/oder mehr als 5 % der eingegliederten Einheiten innerhalb des ersten Jahres fakturieren. Nicht verbrauchte Materialien werden rückerstattet.

15 Monitoring

Ein Monitoring der beteiligten Praxen wird durchgeführt, wenn 10 % der Gesamteinheiten eingegliedert sind. Dabei werden durch den Prüfarzt die Studienunterlagen (Einwilligungserklärungen, Applikations- bzw. Eingliederungsbögen etc.) auf Vollständigkeit geprüft. Queries werden in einem Studientreffen besprochen und ausgeräumt.

16 Data-Safety-Monitoring-Comitée

Die erhobenen Daten werden durch Herrn Prof. W. Hickel als Mitglied des „ Data Safety Monitoring Comitée“ („DSMC“)auf die Richtigkeit geprüft und garantieren, die Richtigkeit der erhobenen Daten im Untersuchungszeitraum von vier Jahren (bei 1+4 Jahren Laufzeit).

17 Berichterstattung

Pro Jahr wird dem Sponsor ein Zwischenbericht eingereicht, der die Ergebnisse des ersten, zweiten und dritten, vierten Studienjahres zusammenfasst. Ein Abschlussbericht erfolgt nach Ablauf des Untersuchungszeitraumes. Regelmäßige Rundbriefe („Newsletter“) der Studienzentrale informieren die Teilnehmer über den aktuellen Stand und auftauchende Probleme der Studiendurchführung.

18 Verantwortlichkeiten und Zuständigkeiten

-LKP: Prof. Dr. Reiner Biffar
OA Dr. Thomas Klinke

-Prüfarzt: OA Dr. Thomas Klinke

- Erstellung des Ethikkommissionsantrages
- Mithilfe bei der biometrischen Planung (Durchführung Dr. Dipl. math. Chr. Schwahn)
- Organisation der Kalibrierung der Kooperationspartnern
- Kontakt mit Kooperationspartnern
- Erarbeitung von SOP’s (*Study-Operating-Procedure*)
- Erstellung des Aufklärungsformulars
- Erstellung des Einverständniserklärung
- Erstellung des Prüfordners
- Randomisierung
- Mitorganisation des Studientreffens mit den Kooperationspartnern
- Erstellung der Zwischenberichte und Rundbriefe
- Erstellung des Abschlussberichtes
- Erstellung einer Publikation

- *Untersuchungsarzt: Kollegen der beteiligten Praxen*

- Führen des Prüfordners und Dokumentation
- Rekrutierung der Probanden
- Terminplanung
- Meldung von unerwünschten Ereignissen
- Datenmanagement mit Kooperationspartnern
- Mitorganisation des Studientreffens

- *Sponsor:*

- Analyse im Schadensfalle
- Einsichtnahme in den Prüfordner
- Übernahme der Kosten (Material, Studienspezifische Applikationshilfen, Mischgeräte etc.)
- Organisation des Studientreffens

18.1 Prüforder

- Votum der Ethikkommission
- Einverständniserklärung des Patienten
- Versicherungsnachweis über den gewährten Versicherungsschutz
- Dokumentation der Behandlungsabfolge

- Anamnese
- Dokumentation der Untersuchungsparameter

    - Zahnarztpraxis:
        - Eingliederung entsprechend des Eingliederungsbogens
        - Nachuntersuchung (1/2-jährlich zur 01-Kontrolle)
        - Anfertigung von Interponaten alle 6 Monate

    - Externer Gutachter:
        - Nachuntersuchung (1x/2a, siehe *Nachuntersuchungsbogen*)

18.2 Studienplan abgestimmt mit Standard

- *Studienspezifisch:*

    - Erhebung der Herstellungsparameter (verantw.: Zahnarztpraxis)
    - Halbjährliche Erfassung der relativen klinischen Parameter (verantw.: Zahnarzt)
    - Jährliche Bestimmung des Zustandes der Restaurationen (verantw.: externer, unabhängiger Gutachter)
    - Schadensanalyse (verantw.: Zahnarzt, OA Dr. Thomas Klinke, Fa. CG)

18.3 Therapieplan abgestimmt mit Standard

- Standarttherapie bei konservierenden/prothetischen Restaurationen

    - Initialtherapie (verantw.: Dentalhygienikerin in der Zahnarztpraxis)
    - Indikationsstellung, Präparation und Applikation (verantw.: Zahnarzt)
    - Bonuskontrolle (verantw.: Zahnarzt)
    - Erhaltungstherapie (im engeren Intervall) (verantw.: Zahnarzt / Dentalhygienikerin)

18.4 Jährliches Follow up organisieren

- Dokumentation bis Studienende bis 60. Monate nach Behandlungsbeginn
- Organisation und Terminierung *(verantw. Praxisteam, GC)*
- Festlegung der Erhaltungstherapie und weiteren Dokumentation

18.5 Info aller Beteiligten

- *Information im jährlichen Follow-up (verantw. GC/Zahnarzt)*

    - Rücksprache mit Prüfarzt, wann Recalltermin stattfinden soll
    - Rücksprache mit Prüfarzt, wenn Patienten Termin absagen oder umbestellen wollen

- *Information im Schadensfall (Kontrolltermin) (verantw. Zahnarzt)*

    - Rücksprache mit Prüfarzt

- *Information der Studienzentrale (verantw. LKP, Prüfarzt)*

    - Rücksprache mit Sponsor

18.6 Rekrutierung der Patienten

- Verantwortlich Zahnarzt und Stellvertreter
- Auswahl aus dem Patientengut der Zahnarztpraxen
- Indikationsstellung f. Rekonstruktion:
  Vitalität, Substanzabtrag
  Die Indikationsstellung richtet sich nach den zahnärztlich-anatomischen Begebenheiten. Dabei ist auf ausreichende, suffiziente Zahnkronendimension und /-konstitution nach der Defektpräparation zu achten.

- Ausschlusskriterien:
    - Risikopatienten
    - CMD-Patienten
    - Füllungen im Frontzahnbereich (außer Kl. V)
    - Patienten mit eingeschränkter Mundhygiene
    - Systematische Parodontalbehandlung innerhalb des vergangenen Jahres

18.7 Randomisierung

- kontrollierte prospektive Überlebensstudie

Jede Praxis erhält einen Randomisierungsbogen, auf dem die zufällige Entscheidung ob ein Patient, der zu einer Versorgung ansteht und bei dem die Indikationen zu treffen, im Aufklärungsgespräch auf die Studie hingewiesen und ihm die Studie erklärt wird. Der Patient entscheidet, ob er an der Studie teilnehmen möchte oder nicht. Sollte der Patient teilnehmen, sollte gewährleistet sein, dass er über die Studiendauer zu den Kontrollterminen erscheint.

18.8 Dokumentation

- Flip-Charts in der Behandlungskarte der Praxen/Abteilung ZZMK
  - Ablaufplan mit Terminen
  - Zeitpunkt der Eingliederung
  - Zeitpunkt des Nachuntersuchungsintervalles
- Erfassung der Ergebnisse der Verlaufsprotokolle im Studienordner
- Erfassung der Taschensondierung (TT), Plaquebefall, Blutungsindex (SBI) im Studienordner
- Erfassung aller Patienten in einer großen tabellarischen Übersicht und farbliche Markierung des Behandlungstandes

18.9 Studientreffen

- Mitorganisation des Studientreffens der Kooperationspartner (verantw. Prüfarzt)
- Bereitstellung der Unterlagen für das Treffen, Organisation Termin, Verschickung der Einladung und Organisation der Bewirtung (verantw. GC)

19 Literatur

Arenholt-Bindsley, D., Kanerva, L.: Die Diagnose von Nebenwirkungen. In: Schmalz, G., Arenholt-Bindsley, D.: Biokompatibilität zahnärztlicher Werkstoffe. Urban & Fischer, München (2005), 349-350.

Bürkle, V., Hickel, R.: Fissurenversiegelung mit Glasionomerzementen - eine Literaturübersicht. Dtsch Zahnärzt Z 2003 (58): 207-211. (Volltext).

British Society of Paediatric Dentistry: a policy document on fissure sealants in paediatric dentistry. International J Pediatr Dent 2000 (10): 174-177. (Volltext, englisch).

Chadwick, B., Dummer, P., Dunstan, F. et al.: How long do fillings last? Evidence-Based Dentistry 2002 (3): 96-99. (Volltext, englisch).

Forss, H., Halme, E.: Retention of glassionomer cement and a resin-based fissure sealant and effect on carious outcome after 7 years. Community Dent Oral Epidemiol 1998 (26): 21. (Zusammenfassung, englisch).

Frankenberger, R., Krämer, N., Petschelt, A.: Werkstoffkundliche Aspekte für ein modernes Behandlungskonzept in der Kinderzahnheilkunde. In: Einwag, J., Pieper, K. (Hrsg.): Kinderzahnheilkunde (Praxis der Zahnheilkunde, Bd. 14), Urban & Fischer, München (2002), 130-133.

Ganesh, M., Shobha, T.: Comparative Evaluation of the Marginal Sealing Ability of Fuji VII and Concise as Pit and Fissure Sealants. J Contemp DEnt Pract 2007 (4): 10-18. (Zusammenfassung, englisch).

Hu, D.Y., Wan, H. C., Liu, H. C., Li, X., Fan, X., Lo, E. C.: Atraumatic restorative treatment restorations placed in school children. Zhonghua Kou Qiang Yi Xue Za Zhi. 2004 Jan;39(1):34-7. (Zusammenfassung, englisch).

Manhart, J., Chen, H. Y., Hickel, R.: Überlebenszeitanalyse von konservierend-zahnärztlichen Restaurationen. Teil II: Direkte Füllungen aus Amalgam und Glasionomerzement im Seitenzahnbereich. ZWR 2003 (5):205-212.

Mejare, I., Mjör, I. A.: Glassionomer and resin-based fissure sealants: a clinical study. Scand J Dent Res 1990 (98): 345. (Zusammenfassung, englisch).

Schmalz, G.: Glasionomer-Zemente. In: Schmalz, G., Arenholt-Bindsley, D.: Biokompatibilität zahnärztlicher Werkstoffe. Urban & Fischer, München (2005), 143-154.

Smales, R. J., Gao, W., Ho, F.: In vitro evaluation of sealing pits and fissures with newer glass-ionomer cements developed for the ART technique. J Clin Pediatr Dent 1997 (21): 321. (Zusammenfassung, englisch).

Qvist, V., Laurberg, L., Poulsen, A., Teglers, P.T.: Eight-year study on conventional glass ionomer and amalgam restorations in primary teeth. Acta Odontol Scand. 2004 Feb;62(1):37-45. (Zusammenfassung, englisch).

Nathanson, D.: No conclusive evidence for caries-inhibitory effect of glass-ionomer restorations in vivo. Evidence-Based Dentistry 2000 (2): 99. (Volltext, englisch).

Setien, V J et al.: Interfacial fracture toughness between resin-modified glass ionomer and dentin using three different surface treatments. Dental Materials 2005; 21: 498-504
Datum: 20.06.2005

20 Anhang

Beispiele für

- Aufklärungsbogen
- Einverständniserklärung des Patienten
- Füllungsprotokoll
- Nachuntersuchungsbogen

20.1 Patientenaufklärung

Sehr geehrte Patientin, sehr geehrter Patient!

Bei der routinemäßigen Untersuchung wurde eine kariöse Stelle im Seitenzahnbereich entdeckt, die mit einer Füllung versorgt werden muss, um weitere Zerstörung des Zahnes aufzuhalten.
Dabei hat die neue Füllung neben der Reparatur der defekten (kariösen) Stelle zwei wesentliche Aufgaben zu erfüllen: sie muss Ihren ästhetischen Ansprüchen genügen, sie muss „unsichtbar" sein und sie muss den Kaukräften widerstehen.
Leider gibt es nicht ein Material, dass allen Ansprüchen auf Dauer entspricht.
Deshalb habe ich mich mit meiner Praxis entschlossen, an einer klinischen Studie teilzunehmen, die von der Universität Greifswald(*) in den kommenden fünf Jahren begleitet werden soll. In dieser Studie soll die Frage beantwortet werden, ob eine neue Materialkombination von dem zahnärztlichen Füllungsmaterial wie Glasionomerzement (Equia®) im Vergleich zu dem gängigen Material (Fuji IX GP®) stabiler ist. Beide dieser Materialien sind zahnfarben und werden im Seitenzahnbereich eingesetzt. Sie sind zugelassene Füllungswerkstoffe, sind hart, widerstehen dem Kaudruck und halten den Zahn kariesfrei.
Damit eine (spätere) Manipulation der Studienergebnisse von vornherein ausgeschlossen ist, entscheidet der Zufall, welches der beiden Materialien bei Ihnen verwendet wird.
In jährlichen Nachuntersuchungen (bis 2014) werden die Füllungen betrachtet und auf Abplatzungen und Brüche (Frakturen) untersucht. Eine kleine Abformung im Zusammenbiss dokumentiert den Verschleiß des Füllungsmaterials.
Drei Mal wird ein eingewiesener Kollege der Universität Greifswald in meiner Praxis erscheinen und die Füllungen ebenfalls begutachten und fotografieren. Für die jährlich stattfindende Untersuchung ist es wichtig, dass Sie sich in meiner Praxis einfinden, damit wir diese Nachuntersuchungen machen können.
Sollte die Füllung unbrauchbar sein oder brechen, wird die Füllung unverzüglich ausgetauscht. Dabei entscheiden Sie, was Sie für ein Füllungsmaterial als Alternative haben möchten. Bei diesem Alternativmaterial (wie z.B. Kunststoff (Composite)) kann unter Umständen durch die aufwendigere Verarbeitung im Vergleich zu dem in der Studie angewendeten Material eine Zuzahlung notwendig werden.
Als Danke-Schön für Ihre Unterstützung dieser Studie erhalten Sie eine Beteiligung in Form von Gutscheinen mit einem Gesamtumfang von bis zu € 70.-- für eine Professionelle Zahnreinigung mit Politur und Fluoridierung in meiner Praxis.

Ich würde mich freuen, wenn Sie als Studienpatient an dieser Studie teilnehmen würden, damit wir gemeinsam diese wichtige wissenschaftliche Frage für die Zahnmedizin beantworten können.

Sollten Sie noch Fragen zu der Untersuchung haben, sprechen Sie mich an.

(*)
Ernst-Moritz-Arndt Universität
Poliklinik für zä. Prothetik, Alterszahnheilkunde und
Medizinische Werkstoffkunde
(Dir.: Prof. Dr. R. Biffar)
Walther-Rathenau-Str. 42a
17475 Greifswald
Tel.: 03834-67140
FAX 03834-867448

20.2 Einverständniserklärung

Probanden-Information und -Einwilligung
zur Durchführung einer klinischen Prüfung eines Medizinproduktes

Prüfstelle: *Poliklinik für zahnärztliche Prothetik, Alterszahnmedizin und Medizinische Werkstoffkunde*

Prüfarzt: OA Dr. Thomas Klinke, Prof. Dr. Reiner Biffar
Poliklinik für Prothetik, Alterszahnmedizin und Med. Werkstoffkunde
Rotgerberstrasse 8, 17487 Greifswald, Tel. 03834 867140

Klinische Bewährung der Füllungsalternative EQUIA

Sehr geehrte Dame, sehr geehrter Herr,

wir fragen an, ob Sie bereit wären, sich mit Ihren geplanten Füllungsversorgungen an einer **klinischen Kontrolle zum Langzeitverhalten** zu beteiligen. Ihr/e Zahnarzt/Zahnärztin ist eine/r unserer ca. 10 Kooperationspartner in Ihrer Stadt. Bei der routinemäßigen Untersuchung durch Ihren Zahnarzt wurde eine kariöse Stelle im Seitenzahnbereich entdeckt, die mit einer Füllung versorgt werden muss, um weitere Zerstörung des Zahnes aufzuhalten. Aufgrund der Einschlusskriterien hat Ihr Zahnarzt/ärztin die Möglichkeit, Sie in die hier beschriebene Studie einzubinden.

Klinische Studien sind in der Zahnmedizin notwendig, um Erkenntnisse über die Haltbarkeit und Widerstandsfähigkeit von Füllungen zu gewinnen oder zu erweitern. Die klinische Studie, die wir Ihnen hier vorstellen, wurde – wie es das Gesetz verlangt – von der zuständigen Ethikkommission zustimmend bewertet. Diese klinische Studie wird in acht Städten Deutschlands durchgeführt; es sollen insgesamt ungefähr 220 Personen daran teilnehmen. Die Studie wurde durch die Universität Greifswald veranlasst und organisiert. Sie wird finanziert durch den Dentalmaterialhersteller GC Europe N.V., Interleuvenlaan 33, B-3001 Leuven, Belgien und GC Germany GmbH, Seifgrundstraße 2, D-61348 Bad Homburg v.d.H., Deutschland, den Sponsor dieser Studie.

Ihre Teilnahme an dieser klinischen Studie ist freiwillig. Sie werden in diese Studie also nur dann einbezogen, wenn Sie dazu schriftlich Ihre Einwilligung erklären. Sofern Sie nicht an der klinischen Studie teilnehmen oder später aus ihr ausscheiden möchten, erwachsen Ihnen daraus keine Nachteile.

Ihr Zahnarzt hat Ihnen bereits eine Reihe von Informationen zu der geplanten Studie gegeben. Der nachfolgende Text soll Ihnen die Ziele und den Ablauf näher erläutern. Anschließend wird ein Aufklärungsgespräch mit Ihnen geführt. Bitte zögern Sie nicht, alle Punkte anzusprechen, die

Ihnen unklar sind. Sie werden danach ausreichend Bedenkzeit erhalten, um über Ihre Teilnahme zu entscheiden.

1. Warum wird diese Studie durchgeführt?

GC Fuji IX GP Extra und Fast, GC G-Coat Plus und LC sind als Medizinprodukte für diesen Einsatzzweck europaweit zugelassen und werden in der Routinebehandlung nach der Entfernung von Karies zum Füllen der Zähne seit längerem eingesetzt und bisher als gleichwertig für diesen Verwendungszweck beurteilt. Sie sind hart, widerstehen dem Kaudruck und halten den Zahn kariesfrei. Die Füllungen haben neben der Reparatur der defekten Stelle im Zahn zwei wesentliche Aufgaben zu erfüllen: sie muss Ihren Ansprüchen genügen und muss den Kaukräften widerstehen. Leider gibt es nicht ein Material, das allen Ansprüchen auf Dauer entspricht. Die Abnutzung und der Bruch von Füllungen sind bekannte und immer wieder auftretende Ereignisse.

In der geplanten klinischen Studie soll über einen Zeitraum von fünf Jahren untersucht werden, ob es Unterschiede im Abnutzungsverhalten und des Füllungsbruches zwischen diesen beiden Produkten des Herstellers gibt. Die beiden Produkte entstammen derselben Materialklasse (Glasionomerzement) und unterscheiden sich nur geringfügig in der Komposition der Bestandteile und ihrer Verarbeitung. Die beschriebene Studie ist **keine Materialprüfung eines neuen Materials**, sondern hier wird die Zuverlässigkeit bekannter Materialien über die Zeitdauer der Studie untersucht.

2. Erhalte ich eines der Prüfpräparate auf jeden Fall?

Im Rahmen dieser klinischen Studie werden zwei bisher als gleichwertig beurteilte Materialien verwendet. Im Falle Ihrer Teilnahme werden Sie entweder GC Equia oder GC Fuji IX GP erhalten**. Welches Füllungsmaterial Sie erhalten, entscheidet ein zuvor festgelegtes Zufallsverfahren, vergleichbar mit dem Werfen einer Münze; dieses wird Randomisierung genannt. Die Wahrscheinlichkeit, ein bestimmtes Material dieser Beiden zu erhalten, beträgt 50 %**.

Zur objektiven Gewinnung von Studiendaten ist es notwendig, dass weder Sie noch Ihr Zahnarzt wissen, welches Präparat ausgewählt wird (dieses Verfahren wird als „doppelblind“ bezeichnet). Sollte es aus Sicherheitsgründen notwendig sein, kann unverzüglich festgestellt werden, welches Präparat Sie erhalten haben. Die Zuordnung ist in einer Liste der Universität Greifswald hinterlegt.

3. Wie ist der Ablauf der Studie und was muss ich bei Teilnahme beachten?

Vor Aufnahme in diese klinische Studie werden Sie zu Familienstand, Schuljahren, höchstem Schulabschluss, dem Empfinden Ihres Gesundheitszustandes der Zähne, Ihren Zahnputzgewohnheiten und Ihrem letzten Zahnarztbesuch befragt.

Bei Teilnahme an der Studie müssen Sie an jeweils einer Kontrolluntersuchung pro Jahr über einen Zeitraum von fünf Jahren teilnehmen. Sie können diese Untersuchung mit der üblichen jährlichen Kontrolluntersuchung gemäß Bonusheft bei Ihrem Zahnarzt kombinieren. Zur Nachkontrolle nach einem, zwei und fünf Jahren wird ein Zahnarzt der Universität Greifswald in der Praxis Ihres Zahnarztes untersuchen. Er wird nur die Füllung/en, die im Rahmen dieser Studie gelegt wurden, auf Abplatzungen und Brüche untersuchen. Zusätzlich wird er zwei kleine Abformungen mit Silikonabformmasse nehmen. Die Abformungen dokumentieren Veränderungen an den Füllungen. Die Untersuchung der restlichen Zähne wird von Ihrem Zahnarzt durchgeführt und steht nicht im Zusammenhang mit dieser Studie. Mit Ihnen wird von Ihrer Zahnarztpraxis ein Termin vereinbart. Bitte haben Sie Verständnis, dass hierfür Termine gesucht werden, an denen alle Teilnehmer an der Studie in der jeweiligen Praxis Ihres Vertrauens teilnehmen können. Es würde uns sehr helfen, wenn Sie die vorgeschlagenen Termine ermöglichen könnten, da ein Untersucher extra für diese Untersuchung von weit anreist. Die Untersuchungen nach drei und vier Jahren werden von Ihrem Zahnarzt eigenständig durchgeführt.

Sollte zwischenzeitlich eine Füllung schadhaft werden, die unter Beobachtung steht, bitten wir Sie, wenn irgend möglich, die Praxis Ihres Zahnarztes aufzusuchen. Sollten Sie dies nicht können, teilen Sie bitte Ihrem Zahnarzt unverzüglich mit, dass eine Füllung schadhaft geworden ist. Da es sich für diesen Zweck um zugelassene Materialien handelt, ist dies ein Ereignis des allgemeinen Risikos, dass auch ohne Beteiligung an der Studie hätte auftreten können.

4. Welchen persönlichen Nutzen habe ich von der Teilnahme an der Studie?

Sie werden durch die Teilnahme an dieser Studie voraussichtlich keinen persönlichen Gesundheitsnutzen haben. Die Ergebnisse der Studie können aber möglicherweise dazu beitragen, die Behandlung mit zahnärztlichen Füllungen und deren Lebensdauer zukünftig weiter zu verbessern.

5. Welche Risiken sind mit der Teilnahme an der Studie verbunden?

Aus der Teilnahme an der Studie entsteht kein anderes oder gar erhöhtes Risiko im Vergleich zu anderen Füllungen dieser Produktkategorie. Spezielle Nebenwirkungen sind bei diesen Produkten nicht bekannt.

6. Wer darf an dieser klinischen Prüfung nicht teilnehmen?

Sie können an dieser klinischen Studie nicht teilnehmen, wenn Sie knirschen und pressen oder unter dauerhaften Muskelbeschwerden der Kaumuskulatur leiden, wenn Sie eine abnehmbare oder Vollprothese tragen und weniger als drei aufeinander beissende Zahnpaare haben.

7. Entstehen für mich Kosten durch die Teilnahme an der klinischen Prüfung? Erhalte ich eine Aufwandsentschädigung?

Durch Ihre Teilnahme an dieser klinischen Prüfung entstehen für Sie keine Kosten.
Für die regelmäßige Teilnahme an der Studie über fünf Jahre erhalten Sie u.a. einen Gesamtzuschuss in Höhe von 70 EUR für die professionelle Zahnreinigung bei Ihrem Zahnarzt.

8. Bin ich während der klinischen Prüfung versichert?

Wir weisen Sie darauf hin, dass Sie auf dem Weg von und zur untersuchenden Zahnarztpraxis versichert sind.
Name und Anschrift der Versicherung:

SV SparkassenVersicherung Gebäudeversicherung AG
Löwentorstraße 65
70376 Stuttgart

**Telefon**:	0180 333 9 333: 9 ct./Min.
**Fax:**	0180 333 9 888: 9 ct./Min............
Versicherungsnummer:	50 025 489 334

9. Werden mir neue Erkenntnisse während der klinischen Prüfung mitgeteilt?

Sie werden über neue Erkenntnisse, die in Bezug auf diese klinische Prüfung bekannt werden und die für Ihre Bereitschaft zur weiteren Teilnahme wesentlich sein können, informiert. Auf dieser Basis können Sie dann Ihre Entscheidung zur weiteren Teilnahme an dieser klinischen Prüfung überdenken.

10. Kann meine Teilnahme an der klinischen Studie vorzeitig beendet werden?

Sie können jederzeit, auch ohne Angabe von Gründen, Ihre Teilnahme beenden, ohne dass Ihnen dadurch irgendwelche Nachteile entstehen.
Unter gewissen Umständen ist es aber auch möglich, dass das Prüfzentrum oder der Sponsor entscheidet, Ihre Teilnahme an der klinischen Studie vorzeitig zu beenden, ohne dass Sie auf die Entscheidung Einfluss haben. Die Gründe hierfür können z. B. im Abbruch der gesamten klinischen Studie liegen.

11. Was geschieht mit meinen Daten?

Während der klinischen Studie werden medizinische Befunde und persönliche Informationen von Ihnen erhoben und in der Prüfstelle in Ihrer persönlichen Akte in der Zahnarztpraxis niedergeschrieben oder elektronisch gespeichert. **Die für die klinische Prüfung wichtigen Daten werden zusätzlich in ausschließlich pseudonymisierter Form gespeichert, ausgewertet und vom Zahnarzt an das Prüfzentrum der Universität Greifswald weitergegeben. Pseudonymisiert bedeutet, dass keine Angaben von Namen oder Initialen verwendet werden, sondern nur ein Nummern- und/oder Buchstabencode, evtl. mit Angabe des Geburtsjahres. Der Schlüssel zum Code verbleibt in der Zahnarztpraxis Ihres Vertrauens und wird von Ihrem Zahnarzt verwaltet**.
Die Daten sind gegen unbefugten Zugriff gesichert. Eine Entschlüsselung erfolgt nur unter den vom Gesetz vorgeschriebenen Voraussetzungen. Daten zur Einbestellungen zu den Kontrolluntersuchungen werden nur in der Zahnarztpraxis Ihres Vertrauens verarbeitet. Sollten Sie der Verwendung der Daten in der beschriebenen Form nicht zustimmen, ist eine Teilnahme an der klinischen Studie nicht möglich.
Einzelheiten, insbesondere zur Möglichkeit eines Widerrufs, entnehmen Sie bitte der Einwilligungserklärung, die im Anschluss an diese Probandeninformation abgedruckt ist.

12. Was geschieht mit meinen Abformungen?

Die Abformungen werden ausschließlich für diese klinische Studie verwendet. Sie werden elektronisch erfasst und unter dem jeweiligen Pseudonym abgelegt.

13. An wen wende ich mich bei weiteren Fragen?

Beratungsgespräche an der Prüfstelle

Sie haben stets die Gelegenheit zu weiteren Beratungsgesprächen mit dem auf Seite 1 genannten oder einem anderen Prüfarzt.

Prüfstelle: *Poliklinik für zahnärztliche Prothetik, Alterszahnmedizin und Medizinische Werkstoffkunde*

Prüfarzt: OA Dr. Thomas Klinke, Prof. Dr. Reiner Biffar
Poliklinik für Prothetik, Alterszahnmedizin und Med. Werkstoffkunde
Rotgerberstrasse 8, 17487 Greifswald, Tel. 03834-867140

Klinische Bewährung der Füllungsalternative EQUIA

Einwilligungserklärung

....
Name des Probanden in Druckbuchstaben

geb. am .... Teilnehmer-Nr. ....

Ich bin in einem persönlichen Gespräch durch den beteiligten Zahnarzt

....
Name der Ärztin/des Arztes

ausführlich und verständlich über die klinische Studie sowie über Wesen, Bedeutung, Risiken und Tragweite der klinischen Studie aufgeklärt worden. Ich habe darüber hinaus den Text der Probandeninformation sowie die hier nachfolgend abgedruckte Datenschutzerklärung gelesen und verstanden. Ich hatte die Gelegenheit, mit dem Prüfarzt über die Durchführung der klinischen Prüfung zu sprechen. Alle meine Fragen wurden zufrieden stellend beantwortet.

Möglichkeit zur Dokumentation zusätzlicher Fragen seitens des Probanden oder sonstiger Aspekte des Aufklärungsgesprächs:

___

___

___

Ich hatte ausreichend Zeit, mich zu entscheiden.

Mir ist bekannt, dass ich jederzeit und ohne Angabe von Gründen meine Einwilligung zur Teilnahme an der Prüfung zurückziehen kann (mündlich oder schriftlich), ohne dass mir daraus Nachteile entstehen.

Datenschutz:

Mir ist bekannt, dass bei dieser klinischen Studie personenbezogene Daten, insbesondere medizinische Befunde über mich erhoben, gespeichert und ausgewertet werden sollen. Die Verwendung der Angaben über meine Gesundheit erfolgt nach gesetzlichen Bestimmungen und setzt vor der Teilnahme an der klinischen Prüfung folgende freiwillig abgegebene Einwilligungserklärung voraus, das heißt ohne die nachfolgende Einwilligung kann ich nicht an der klinischen Prüfung teilnehmen.

1. Ich erkläre mich damit einverstanden, dass im Rahmen dieser klinischen Studie personenbezogene Daten, insbesondere Angaben über meine Gesundheit, über mich erhoben und in Papierform sowie auf elektronischen Datenträgern bei meinem Zahnarzt aufgezeichnet werden. Die erhobenen Daten werden pseudonymisiert (verschlüsselt) an das Prüfzentrum in Greifswald weitergegeben werden:

   a) an das Prüfzentrum, den Sponsor oder eine von diesem beauftragte Stelle zum Zwecke der wissenschaftlichen Auswertung,

   c) im Falle unerwünschter Ereignisse: an das Prüfzentrum, den Sponsor, an die jeweils zuständige Ethik-Kommission und die zuständige Bundesoberbehörde *Bundesinstitut für Arzneimittel und Medizinprodukte*, sowie von dieser an die Europäische Datenbank.

2. Außerdem erkläre ich mich damit einverstanden, dass autorisierte und zur Verschwiegenheit verpflichtete Beauftragte des Sponsors sowie die zuständigen Überwachungsbehörden in meine beim Zahnarzt vorhandenen personenbezogenen Daten, insbesondere meine Gesundheitsdaten, Einsicht nehmen, soweit dies für die Überprüfung der ordnungsgemäßen Durchführung der Studie notwendig ist. Für diese Maßnahme entbinde ich den Zahnarzt und die Prüfärzte des Prüfzentrums von der ärztlichen Schweigepflicht.

3. Die Einwilligung zur Erhebung und Verarbeitung meiner personenbezogenen Daten, insbesondere der Angaben über meine Gesundheit, ist unwiderruflich. Ich bin bereits darüber aufgeklärt worden, dass ich jederzeit die Teilnahme an der klinischen Studie beenden kann. Im Fall eines solchen Widerrufs meiner Einwilligung, an der Studie teilzunehmen, erkläre ich mich damit einverstanden, dass die bis zu diesem Zeitpunkt gespeicherten Daten weiterhin verwendet werden dürfen, um die ausreichende Power der Studie zu erhalten.

4. Ich erkläre mich damit einverstanden, dass meine Daten nach Beendigung oder Abbruch der Studie mindestens zehn Jahre aufbewahrt werden, wie es die Vorschriften über die klinische Prüfung von Medizinprodukten bestimmen. Danach werden meine personenbezogenen Daten gelöscht, soweit nicht gesetzliche und satzungsmäßige Aufbewahrungsfristen entgegenstehen.

5. Ich bin über folgende gesetzliche Regelung informiert: Falls ich meine Einwilligung, an der Studie teilzunehmen, widerrufe, müssen alle Stellen, die meine personenbezogenen Daten, insbesondere Gesundheitsdaten, gespeichert haben, unverzüglich prüfen, inwieweit die gespeicherten Daten für die in Nr. 3 genannten Zwecke noch erforderlich sind.

   Nicht mehr benötigte Daten sind unverzüglich zu löschen.

Ich erkläre mich bereit,

an der oben genannten klinischen Prüfung

freiwillig teilzunehmen.

Ein Exemplar der Probanden-Information und -Einwilligung habe ich erhalten. Ein Exemplar verbleibt im Prüfzentrum der Poliklinik für Prothetik, Alterszahnmedizin und Med. Werkstoffkunde.

....
Name des Probanden in Druckbuchstaben

....
Datum Unterschrift des **Probanden**

Ich habe das Aufklärungsgespräch geführt und die Einwilligung des Probanden eingeholt.

....
Name des Zahnarztes/-ärztin in Druckbuchstaben

....
Datum

20.3 Beispiel für Füllungsprotokoll

Nach dem Legen der Füllung tagen Sie bitte die Probandennummer und das Geburtsdatum des Probanden sowie das Eingliederungsdatum und die/den versorgten Zahn/Zähne in die u.a. Liste ein.

Umschlag	Probandennr	Geburtsdat.	Eingliederungsdat.	Versorgte/r Zahn/Zähne
**Beispiel**	*# 3456*	*1.2.1965*	*21.6.2009*	*15o, 16od*
**1**				
**2**				
**3**				
**4**				
**5**				
**6**				
**7**				
**8**				

Praxisstempel

QM-Management:

Farbe A 3,5
Farbe Gelb:
LOT 0906011
EXP XXX-XXX
CE 0086

Farbe weiß:
LOT 0901061
EXP. XXXX-XX
CE 0086

20.4 Beispiel für Nachuntersuchungsbogen

EQUIA Follow Up NUXXX

**Praxis:**	
Untersuchungsdatum:	
Beh. Nr. Pat	Geb.Datum
Bildnummern: Interponat: Replika:	

Versorgung	1	2
Zahn		
Flächen	☐ F_1<br>☐ F_2 Fläche:_________	☐ F_1<br>☐ F_2 Fläche: ________
A1<br>Oberflächenglanz	☐ Schmelzart. Glanz,<br>☐ matte Oberfl., unauffällig<br>☐ matte Oberfl., isolierte Porositäten<br>☐ matte , akzeptable Oberfl. (Speichelf.)<br>☐ multiple Porositäten (<1/3 d. Oberfl.)<br>☐ raue Oberfl, Politur unzur., Neuanfert.<br>☐ raue Oberfl. (Hohlräume)<br>☐ sehr, raue, plaqueretent. Oberfl.	☐ Schmelzart. Glanz,<br>☐ matte Oberfl., unauffällig<br>☐ matte Oberfl., isolierte Porositäten<br>☐ matte , akzeptable Oberfl. (Speichelf.)<br>☐ multiple Porositäten (<1/3 d. Oberfl.)<br>☐ raue Oberfl, Politur unzur., Neuanfert.<br>☐ raue Oberfl. (Hohlräume)<br>☐ sehr, raue, plaqueretent. Oberfl.
B5<br>Materialfraktur und Retention	☐ keine Haarrisse o. Frakturen<br>☐ schmale Haarrisse<br>☐ zwei/mehr größ. Risse/Abpl.o.Randb.<br>☐ Abplatzung (Chip) i.Kontaktbereich<br>☐ größ. Abplatz.< ½ d. Flgsfl.<br>☐ teilw. Verlust / multiple Frakturen	☐ keine Haarrisse o. Frakturen<br>☐ schmale Haarrisse<br>☐ zwei/mehr größ. Risse/Abpl.o.Randb.<br>☐ Abplatzung (Chip) i.Kontaktbereich<br>☐ größ. Abplatz.< ½ d. Flgsfl.<br>☐ teilw. Verlust / multiple Frakturen
B6<br>Marginale Adaptation	☐ harm. Gestaltg, keine weißl .Lin/Spalt<br>☐ marg. Spalt (<150µm), weißl.Linie<br>☐ schmale, marg.Frakt. (Polit.notwend)<br>☐ ger. Stufe, minim. Unregelmäßigkt.<br>☐ Stufe < 250µm, nicht korrigierbar<br>☐ einige marginale Frakturen<br>☐ größere Abplatzungen<br>☐ Stufe > 250µm, Dentin/UF exponiert<br>☐ einige marginale Abplatzungen<br>☐ größere Stufe (Neuanf.notwendig)<br>☐ Retentionsverlust (Flg. in-situ)<br>☐ generalisierte, größere Spalten	☐ harm. Gestaltg, keine weißl .Lin/Spalt<br>☐ marg. Spalt (<150µm), weißl.Linie<br>☐ schmale, marg.Frakt. (Polit.notwend)<br>☐ ger. Stufe, minim. Unregelmäßigkt.<br>☐ Stufe < 250µm, nicht korrigierbar<br>☐ einige marginale Frakturen<br>☐ größere Abplatzungen<br>☐ Stufe > 250µm, Dentin/UF exponiert<br>☐ einige marginale Abplatzungen<br>☐ größere Stufe (Neuanf.notwendig)<br>☐ Retentionsverlust (Flg. in-situ)<br>☐ generalisierte, größere Spalten
B7<br>Occlusale Kontur und Abstützung	☐ phys. Abstützung auf Schmelzniveau<br>☐ Abstützung 80-120% d. Schmelzes<br>☐ normale Abst., klein Diff.z.Schmelz<br>☐ 50-80%/120-150% Abnutz.z.Schmelz<br>☐ normale Abstützg i.physiolog. Varianz<br>☐ Abnutzg <50%/150-300% d. Schm.niv<br>☐ betr.Abnutzg. den Schm.überrag./Kpktverlust<br>☐ Abnutzung < 300% zum Antagonist<br>☐ überhöhte Abnutzung<br>☐ Abnutzung < 500% zum Antagonist	☐ phys. Abstützung auf Schmelzniveau<br>☐ Abstützung 80-120% d. Schmelzes<br>☐ normale Abst., klein Diff.z.Schmelz<br>☐ 50-80%/120-150% Abnutz.z.Schmelz<br>☐ normale Abstützg i.physiolog. Varianz<br>☐ Abnutzg <50%/150-300% d. Schm.niv<br>☐ betr.Abnutzg. den Schm.überrag./Kpktverlust<br>☐ Abnutzung < 300% zum Antagonist<br>☐ überhöhte Abnutzung<br>☐ Abnutzung < 500% zum Antagonist
B8<br>Approximale anatomische Form	☐ normaler Kontaktpkt, 25µm Band<br>☐ normale Kontur<br>☐ Kontaktpkt stark, n. nachteilg, 25µm Presspassg<br>☐ leicht unzur. Kontur<br>☐ schwacher Kontaktpkt, 50µm Band<br>☐ sichtb. unzur. Kontur<br>☐ Kontaktpkt zu schwach, Parod.schäden, 100µm Band<br>☐ inadäquat. Kontur, Papillenenzündung<br>☐ insuff. Kontur, erneuerungswürdig	☐ normaler Kontaktpkt, 25µm Band<br>☐ normale Kontur<br>☐ Kontaktpkt stark, n. nachteilg, 25µm Presspassg<br>☐ leicht unzur. Kontur<br>☐ schwacher Kontaktpkt, 50µm Band<br>☐ sichtb. unzur. Kontur<br>☐ Kontaktpkt zu schwach, Parod.schäden, 100µm Band<br>☐ inadäquat. Kontur, Papillenenzündung<br>☐ insuff. Kontur, erneuerungswürdig

© 2010, Th. Klinke, Ernst-Moritz-Arndt-Universität Greifswald
nach Hickel et al: FDI: Clinical criteria for evaluation of direct and indirect Restorations
Clin Oral Invest (2010) 14:349-366

B10<br>Patienten Einschätzung	☐ vollkom. Zufriedenheit (Ästhetik/Funktion)<br>☐ befriedigend ☐ befried. Ästhetik<br>☐ befried. Funktion, minimale Rauhigkeit<br>☐ minimale Kritik, keinen klinischen Effekt<br>☐ ästh. Mängel ☐ einige Kaumängel<br>☐ unangenehme Versorgungsprozedur<br>☐ Nachbesserung erwünscht ☐ Ästhetik<br>☐ Funktion, Zungenirrit. Nachbesserung anat. Form/Politur möglich<br>☐ voll. Unzufriedenheit, nacht. Effekt, Schmerzen	☐ vollkom. Zufriedenheit (Ästhetik/Funktion)<br>☐ befriedigend ☐ befried. Ästhetik<br>☐ befried. Funktion, minimale Rauhigkeit<br>☐ minimale Kritik, keinen klinischen Effekt<br>☐ ästh. Mängel ☐ einige Kaumängel<br>☐ unangenehme Versorgungsprozedur<br>☐ Nachbesserung erwünscht ☐ Ästhetik<br>☐ Funktion, Zungenirrit. Nachbesserung anat. Form/Politur möglich<br>☐ voll. Unzufriedenheit, nacht. Effekt, Schmerzen
C11<br>Postoperative Sensibilität und Vitalität	☐ keine Hypersensibilität, Vitalität vorhanden<br>☐ geringf. Hypersensibilität f.kurz.Zeitraum<br>☐ milde, versp. Hypersensibilität, kein Reklam.<br>☐ intensive Hypersensibilität<br>☐ verzögerte Sensibilität m ger. Symptomen<br>☐ keine Sensibilität messbar<br>☐ akute Pulpitis, Devitalität, Endo notwendig	☐ keine Hypersensibilität, Vitalität vorhanden<br>☐ geringf. Hypersensibilität f.kurz.Zeitraum<br>☐ milde, versp. Hypersensibilität, kein Reklam.<br>☐ intensive Hypersensibilität<br>☐ verzögerte Sensibilität m ger. Symptomen<br>☐ keine Sensibilität messbar<br>☐ akute Pulpitis, Devitalität, Endo notwendig
C12<br>Materialinteraktion mit dem Zahn und Zahnhalteapparat	☐ keine Primär-/Sekundaärkaries<br>☐ kleine Demineralisierung<br>☐ kleine Erosionen<br>☐ kleine lokale Aussprengungen<br>☐ größere Demineralisation<br>☐ größere Erosionen<br>☐ größere Absprengungen, keine Dentinexposition, Fluoridierung notwendig<br>☐ Karieskavitation / unterminierende Karies<br>☐ Erosionen im Dentin<br>☐ Absprengung m. Dentinexposition<br>☐ Tiefe Karies, keine Reparaturmöglichkeit	☐ keine Primär-/Sekundärkaries<br>☐ kleine Demineralisierung<br>☐ kleine Erosionen<br>☐ kleine lokale Aussprengungen<br>☐ größere Demineralisation<br>☐ größere Erosionen<br>☐ größere Absprengungen, keine Dentinexposition, Fluoridierung notwendig<br>☐ Karieskavitation / unterminierende Karies<br>☐ Erosionen im Dentin<br>☐ Absprengung m. Dentinexposition<br>☐ Tiefe Karies, keine Reparaturmöglichkeit
C13<br>Zahnintaktheit<br>„Tooth interity“	☐ komplette Unversehrtheit<br>☐ kleine marg. Schmelzfrakturen (<150µm)<br>☐ Haarrisse im Schmelz (<150µm)<br>☐ marg. Schmelzdefekte (<250µm)<br>☐ Schmelzrisse (<250µm)<br>☐ multiple Schmelzrisse<br>☐ größere Schmelzdefekte >250µm, Dentinexp.<br>☐ größere Schmelzdefekte >250µm, sondierbar<br>☐ große Schmelzabsprengung, Wandfrakturen<br>☐ Höcker-/Zahnfraktur	☐ komplette Unversehrtheit<br>☐ kleine marg. Schmelzfrakturen (<150µm)<br>☐ Haarrisse im Schmelz (<150µm)<br>☐ marg. Schmelzdefekte (<250µm)<br>☐ Schmelzrisse (<250µm)<br>☐ multiple Schmelzrisse<br>☐ größere Schmelzdefekte >250µm, Dentinexp.<br>☐ größere Schmelzdefekte >250µm, sondierbar<br>☐ große Schmelzabsprengung, Wandfrakturen<br>☐ Höcker-/Zahnfraktur
