# Supplementary material for: Dental practitioner recruitment for a randomized clinical trial in the field to evaluate the performance of a new glass ionomer restoration material
Source: Trials. 2016 Feb 10;17:73. doi: 10.1186/s13063-016-1198-3 (PMC4748549; doi:10.1186/s13063-016-1198-3)
Supplement: Additional file 5: — CONSORT flow diagram. (DOC 51 kb) [file 13063_2016_1198_MOESM5_ESM.doc]

**
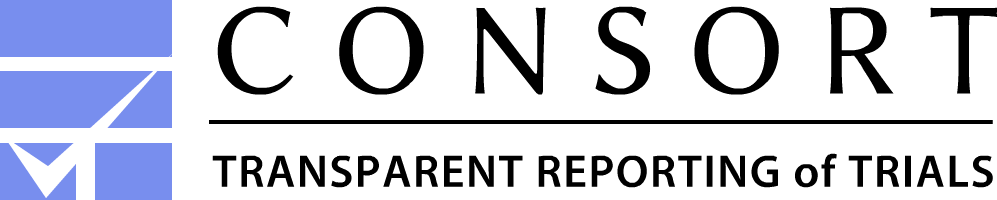
**

**CONSORT 2010 Flow Diagram**

**Allocation**

**Analysis**

**Enrollment**

Randomized written letter of invitation, (n=3194)

Excluded (n= 2959)

- Adverse decision (n=1609)
- No decision (n=1350)

Analysis of socio-epidemical status, practice [specialization](http://www.dict.cc/englisch-deutsch/specialization.html) and specification (n=144)

♦ Participation GCP seminar, signed study agreement (n=144)

Promised participation, Invitation for GCP seminar (n=235)

Excluded (n=91)

- Agreed to participate but didn’t show up, no information given

Study termination, (n=14)
Lost to follow up
